# The Influence of PVP Polymer Topology on the Liquid Crystalline Order of Itraconazole in Binary Systems

**DOI:** 10.1021/acs.molpharmaceut.4c00215

**Published:** 2024-05-17

**Authors:** Luiza Orszulak, Taoufik Lamrani, Roksana Bernat, Magdalena Tarnacka, Daniel Żakowiecki, Karolina Jurkiewicz, Patryk Zioła, Anna Mrozek-Wilczkiewicz, Andrzej Zięba, Kamil Kamiński, Ewa Kamińska

**Affiliations:** † Institute of Chemistry, Faculty of Science and Technology, University of Silesia in Katowice, Szkolna 9, 40-007 Katowice, Poland; ‡ Institute of Physics, Faculty of Science and Technology, University of Silesia in Katowice, 75 Pulku Piechoty 1A, 41-500 Chorzow, Poland; § Department of Pharmacognosy and Phytochemistry, Faculty of Pharmaceutical Sciences in Sosnowiec, Medical University of Silesia in Katowice, Jagiellonska 4, 41-200 Sosnowiec, Poland; ∥ Institute of Materials Engineering, Faculty of Science and Technology, University of Silesia in Katowice, 75 Pulku Piechoty 1A, 41-500 Chorzow, Poland; ⊥ Chemische Fabrik Budenheim KG, Rheinstrasse 27, 55257 Budenheim, Germany; # Biotechnology Centre, Silesian University of Technology, Boleslawa Krzywoustego 8, 44-100 Gliwice, Poland; ∇ Department of Organic Chemistry, School of Pharmacy with the Division of Laboratory Medicine in Sosnowiec, Medical University of Silesia in Katowice, Jagiellonska 4, 41-200 Sosnowiec, Poland

**Keywords:** itraconazole, poly(vinylpyrrolidone), star-shaped polymer, binary mixtures, amorphous solid dispersions, nematic phase, smectic phase, liquid crystalline order

## Abstract

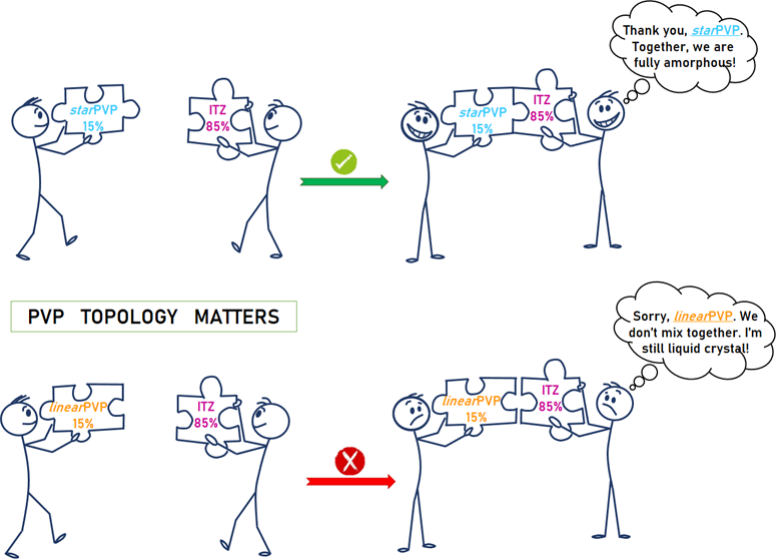

This study presents a novel approach by utilizing poly(vinylpyrrolidone)s (PVPs) with various topologies as potential matrices for the liquid crystalline (LC) active pharmaceutical ingredient itraconazole (ITZ). We examined amorphous solid dispersions (ASDs) composed of ITZ and (*i*) self-synthesized linear PVP, (*ii*) self-synthesized star-shaped PVP, and (*iii*) commercial linear PVP K30. Differential scanning calorimetry, X-ray diffraction, and broad-band dielectric spectroscopy were employed to get a comprehensive insight into the thermal and structural properties, as well as global and local molecular dynamics of ITZ–PVP systems. The primary objective was to assess the influence of PVPs’ topology and the composition of ASD on the LC ordering, changes in the temperature of transitions between mesophases, the rate of their restoration, and finally the solubility of ITZ in the prepared ASDs. Our research clearly showed that regardless of the PVP type, both LC transitions, from smectic (Sm) to nematic (N) and from N to isotropic (I) phases, are effectively suppressed. Moreover, a significant difference in the miscibility of different PVPs with the investigated API was found. This phenomenon also affected the solubility of API, which was the greatest, up to 100 μg/mL in the case of *star*PVP 85:15 *w/w* mixture in comparison to neat crystalline API (5 μg/mL). Obtained data emphasize the crucial role of the polymer’s topology in designing new pharmaceutical formulations.

## Introduction

The pharmaceutical industry is a rapidly evolving sector of the economy. However, despite its dynamic development, bringing new pharmaceutical products to the market encounters several serious problems. One of them is the very poor solubility of a large number of active pharmaceutical ingredients (APIs) and, consequently, their low bioavailability.^1^ Hence, in recent years, many scientific groups have been working on various strategies to increase the solubility and therapeutic effectiveness of APIs.^2−4^ In this context, one can mention several methods, e.g., salt formation,^5^ cocrystallization,^6,7^ micronization,^8^ or amorphization.^9^ The latter technique, i.e., the transformation of the crystalline active substance into the fully disordered (amorphous) form, is a highly promising research direction.^4^ Numerous experimental and theoretical studies have indicated that amorphous pharmaceuticals due to higher entropy and free energy than their crystalline counterparts exhibit greater solubility and bioavailability.^10^ On the other hand, the amorphous phase is thermodynamically unstable, which creates a high possibility of spontaneous crystallization under storage and processing conditions.^11,12^ Nevertheless, this example clearly shows that it is quite easy to enhance both the physicochemical and pharmacokinetic properties of pharmaceuticals by a simple manipulation of the degree of molecular order/disorder. Considering this fact, special attention is paid to looking for materials characterized by various kinds of molecular ordering, which can be tuned by employing different external factors, such as pressure, temperature, solvent, pH, etc. Among all studied systems until now, liquid crystals (LCs) are looming as the best candidates, satisfying the above criteria.^13^ These materials exhibit features of both three-dimensionally ordered solid crystals and conventional liquids^14^ and are characterized by the abundance of phases differing in structural order. Two of the most common liquid crystalline (LC) mesophases are the nematic (N) and smectic (Sm) phases. In the former, rodlike molecules are arranged parallel to each other, with their long axes pointing in approximately the same direction. At the same time, their centers of mass are arranged randomly; thus, only the orientational order is maintained. In the Sm phase, the molecules located within layers are arranged in planes—hence, besides the orientational order, there is also a positional order.^14−16^ Moreover, it should be emphasized that there are two main types of LCs: thermotropic and lyotropic liquid crystals (TLCs and LLCs), where LC phases can be induced by changing the temperature (heating or cooling) or dissolving mesogens in a suitable solvent, respectively.^13^ Therefore, it is possible to obtain various states of molecular order. Consequently, due to the change in the spatial organization of molecules and higher Gibbs energy, the dissolution rate and bioavailability of LC APIs can be optimized.^10,14,17−19^ Hence, studies aimed at in-depth characterization of pharmaceuticals forming LC phases are entirely justified.

Certainly, among thermotropic LC APIs, an extremely interesting example is itraconazole (ITZ). It is a broad-spectrum antifungal medication belonging to the triazole antifungals.^20,21^ According to the biopharmaceutics classification system, ITZ is considered a class II drug due to its extremely low aqueous solubility (merely 1 ng/mL), which increases to 5 μg/mL in an acid solution.^22^ The first measurements of ITZ using differential scanning calorimetry (DSC) were performed by Six et al.^23^ They observed two additional endothermic phase transitions in thermograms, at *T* = 346 and 363 K, located above the glass-transition temperature (*T*_g_ = 332 K). The peak occurring at 363 K was interpreted as the transition from the isotropic liquid to the chiral nematic mesophase, while the transition at a lower *T* (= 346 K) was considered as most likely caused by a restriction in the rotation of molecules. Further studies on the molecular dynamics of ITZ carried out by Tarnacka et al.^24^ clearly indicated that the observed endothermic events are indeed associated with the formation of the nematic N (363 K) and smectic Sm (346 K) phases. Since that time, ITZ has been thoroughly studied by different research groups.^25−34^ Furthermore, new reports still continue to emerge, shedding light on their remarkably intriguing LC properties and the possibilities for their modification. Herein, one can mention a paper by Teerekapibal et al., who investigated the influence of the cooling rate (ϕ) of liquid ITZ on the occurrence of mesophases.^30^ The experiments have demonstrated that when ϕ is equal to 20 K/s, the transition from N to Sm phase is completely suppressed, while at the standard ϕ (10 K/min), the latter phase is formed. Moreover, a recent study by Heczko et al. has shown that the rapid compression of liquid API has an effect similar to fast cooling—an effective suppression of the N–Sm transition.^26^ As a result, in both cases (rapid cooling and compression), only the nematic order was preserved in the glassy phase. Another interesting approach to tuning molecular order in ITZ was presented by Ediger’s group, where the authors in a series of articles indicated that by varying temperature and the rate of vapor deposition of ITZ on a substrate, one can modify the molecular arrangement in this system.^31,32^ Finally, it should be mentioned that Knapik-Kowalczuk et al.^33^ and Mierzwa et al.^34^ demonstrated that another external force, i.e., mechanical shearing, influences ITZ spatial alignment.

Herein, it is worth noting that apart from the physical factors affecting the LC ordering of ITZ, the role of the addition of low- and high-molecular-weight compounds is extensively studied. In the paper by Kaminska et al., it was demonstrated that in the ITZ-acetylated maltose amorphous solid dispersions (ASDs) with various API contents, both LC transitions are effectively suppressed.^35^ In another work, Amponsah-Efah et al. reported that the addition of glycerol has a significant effect on the phase behavior of this LC pharmaceutical. Interestingly, they observed a spectacular change in the phase sequence and the order of phase transitions in the considered ITZ-glycerol binary mixture.^36^ Another intriguing research showed the impact of various konazoles on the LC properties of ITZ. The authors indicated that with an increasing content of three of the used APIs, i.e., ketonazole, fluconazole, and voriconazole, both the Sm and N ordering in ITZ are suppressed.^37^ Furthermore, there are reports depicting the effect of polymer addition on the LC order of ITZ. Cruz et al. revealed that the type of poly(ethylene glycol) used has a significant impact on the suppression or enhancement of the Sm phase in API particles, thereby contributing to the improvement or deterioration of the drug’s solubility, respectively.^38^ It is also worth noting that in the majority of articles addressing formulations of API–polymer systems, researchers predominantly used commercially available macromolecules. In particular, there are many works focused on ASDs of ITZ with Soluplus (poly(vinyl caprolactam)–poly(vinyl acetate)–poly(ethylene glycol) graft copolymer),^39^ KollidonVA64 (vinylpyrrolidone–vinyl acetate copolymer),^40^ Kollicoat IR (polyethylene glycol–poly(vinyl alcohol) graft copolymer),^41^ or EudiragitE100 (poly(methacrylate)s derivative).^40^ However, in the mentioned papers, the authors noted only that polymers partially suppress the Sm and/or N order of ITZ but do not cause their complete and permanent disappearance. Only DiNunzio et al. showed that the preparation of fully amorphous binary mixtures of ITZ with poly(vinyl acetate phthalate) by ultrarapid freezing leads to a significant improvement in ITZ bioavailability. Unfortunately, in this work, the authors did not discuss whether LC phases reconstruct with time.^42^

Furthermore, in the literature, there are no reports that show directly how the polymer structure affects the solubility and LC ordering of ITZ. There is also a general interest in understanding how other macromolecules (beyond commercial polymers) influence the formation of mesophases in this API. Thus, to cover this intriguing research gap, we performed pioneering studies on the impact of poly(vinylpyrrolidone) (PVP) topology on the disruption/suppression of the LC order of ITZ, a possible reconstruction of smectic or nematic order, as well as the solubility of this active substance.

## Materials and Methods

### Materials

1-Vinyl-2-pyrrolidone (VP, >99%, Sigma-Aldrich, Poznan, Poland) was passed through an alumina column before use to remove the inhibitor. 2,2′-Azobis(2-methylpropionitrile) solution (AIBN, 0.2 M in toluene, Sigma-Aldrich, Poznan, Poland), cyanomethyl methyl(4-pyridyl) carbamodithioate (CTA1, 98%, Sigma-Aldrich, Poznan, Poland), 1,3,5-tris(bromomethyl)benzene (97%, Sigma-Aldrich, Poznan, Poland), sodium diethyldithiocarbamate trihydrate (Sigma-Aldrich, Poznan, Poland), diethyl ether (pure for analysis, Chempur, Piekary Slaskie, Poland), methanol (99.85%, PureLand, Stargard, Poland), dichloromethane (DCM, 99%, Karpinex, Warszawa, Poland), chloroform-*d* (99.8% D, contains 0.03% *v/v* TMS, Sigma-Aldrich, Poznan, Poland), PVP K30 (*M_n_* = 40 500, *Đ* = 2.05, Sigma-Aldrich, Poznan, Poland), and neat ITZ (IUPAC name: (2*R*,4*S*)- rel-1-(butan-2-yl)-4-{4-[4-(4-{[(2*R*,4*S*)-2-(2,4-dichlorophenyl)-2-(1*H*1,2,4-triazol-1-ylmethyl)-1,3-dioxolan-4yl]methoxy}phenyl)piperazin-1-yl]phenyl}-4,5-dihydro-1*H*-1,2,4-triazol-5-one, 99%, Sigma-Aldrich, Poznan, Poland) were used as received. The polymorphic form of the commercial ITZ was the stable form I (triclinic space group P1 with 4 molecules per unit cell and lattice parameters: *a* ≈ 8.62 Å, *b* ≈ 20.15 Å, *c* ≈ 21.04 Å, α ≈ 73.49°, β ≈ 89.07°, γ ≈ 79.78°).^43,44^ The same polymorphic form was observed after the recrystallization of ITZ from binary mixtures with PVP.

### Procedure of Preparing ITZ–PVP Mixtures

**Figure 1:**
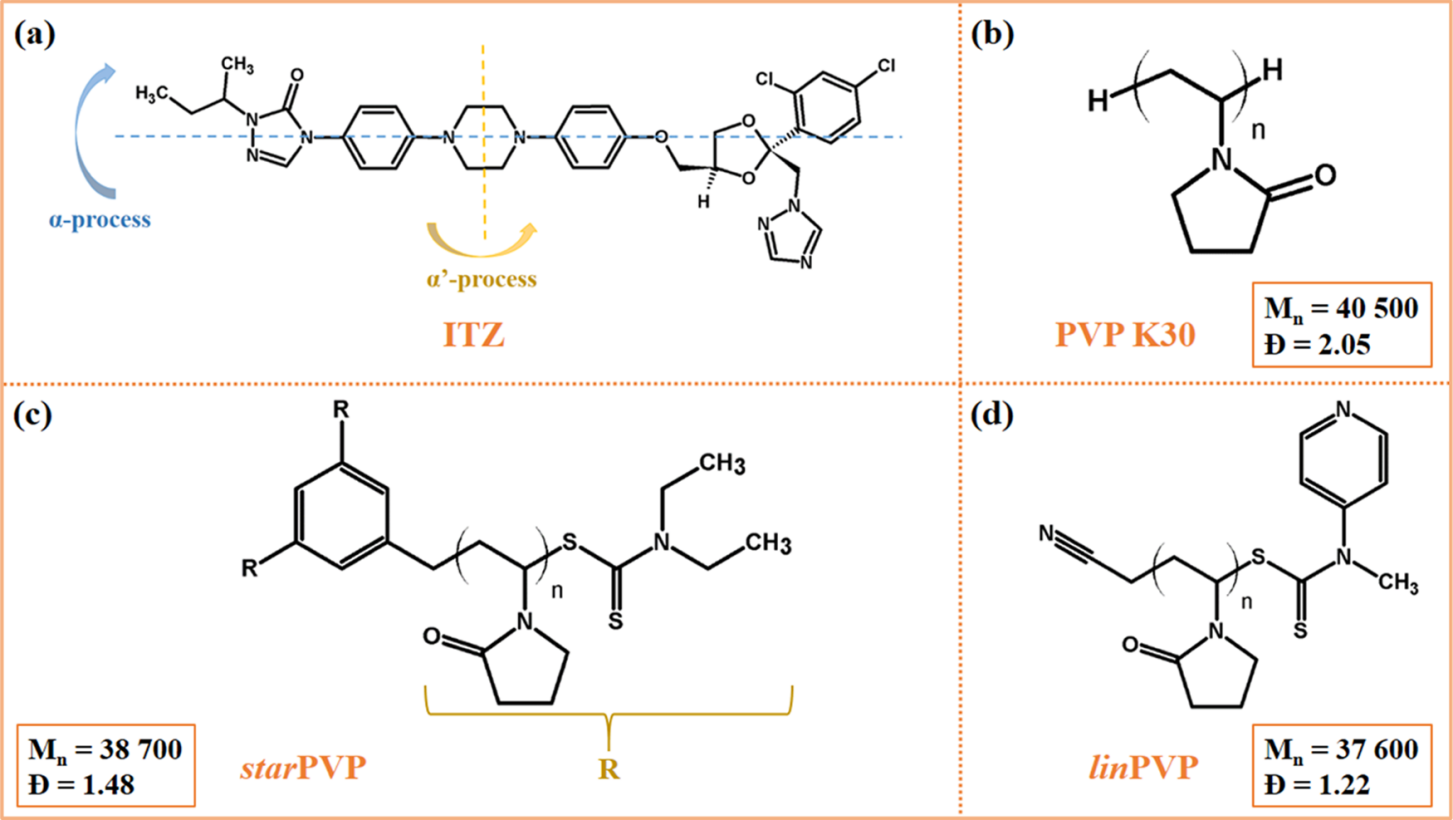
Chemical structure of (a) ITZ with marked motions being the source of two relaxation processes (α and α′), (b) commercially available PVP K30, (c) self-synthesized *star*PVP, and (d) self-synthesized *lin*PVP.

Amorphous solid dispersions (ASDs) composed of ITZ and PVP polymers, including a commercial PVP K30, self-synthesized linear, and star-shaped PVP samples (see Figure 1), were obtained using the melt-cooling method. The ITZ–PVP systems were prepared at the same weight ratio of API to polymer, i.e., 95:5 *w/w*. In the case of ITZ–*star*PVP, the 90:10 and 85:15 *w/w* systems were also examined. To determine a homogeneous mixture, appropriate amounts of neat ITZ and PVP were weighed, carefully transferred to a metal plate, and preliminarily mixed using a spatula. Subsequently, the plate with the API–polymer system was transferred to a hot plate heated to a temperature of 453 K. After a while, ITZ began to melt and the entire binary mixture was stirred until the complete mixing of PVP in the API. Once homogeneity of the system was ensured, the sample was vitrified by rapidly transferring it to a precooled copper substrate. Herein, it is worth noting that the same melt-cooling procedure was used to obtain the amorphous form of neat ITZ.

### Fourier-Transform Infrared Spectroscopy (FTIR)

FTIR spectra were collected on a Nicolet iS50 FTIR spectrometer (Thermo Scientific) with a built-in diamond iS50 ATR sampling station in the range of 4000–400 cm^–1^ with a resolution of 4 cm^–1^. Sixteen scans were averaged for each spectrum.

### Differential Scanning Calorimetry (DSC)

Differential scanning calorimetry measurements were performed using a Mettler–Toledo DSC apparatus (Mettler–Toledo International, Inc., Greifensee, Switzerland) equipped with a liquid nitrogen cooling accessory and an HSS8 ceramic sensor (heat flux sensor with 120 thermocouples). Temperature and enthalpy calibrations were performed using indium and zinc standards. All investigated samples (neat PVP polymers and ITZ–PVP, 95:5 and 85:5 *w/w* systems) were scanned at a rate of 10 K/min over a temperature range from 298 to 480 K. Moreover, additional DSC studies at heating rates of 5 and 20 K/min were conducted for ITZ–PVP 95:5 *w/w* mixtures.

### X-ray Diffraction (XRD)

X-ray diffraction experiments were carried out using a Rigaku D/Max Rapid II diffractometer equipped with a rotating Ag anode and an incident beam (002) graphite monochromator (wavelength of the incident beam λ = 0.56 Å). Samples were measured in borosilicate glass capillaries with the Debye–Scherrer geometry. The diffraction patterns were collected in a single shot using a two-dimensional (2D) image plate detector and transferred to the function of the scattering vector *Q =* 4π* *sin θ/λ, where 2θ is the scattering angle. The background intensity from the empty capillary was subtracted from the data collected for the samples. The temperature during the measurements was 298 K. The samples were closed and long-term stored in capillaries at 323 K. Before XRD measurements, each sample was dried in a vacuum desiccator for at least 1 h to remove any moisture.

### Broad-Band Dielectric Spectroscopy (BDS)

Isobaric measurements of the dielectric permittivity were performed using the Novo-Control α dielectric spectrometer (Novocontrol Technologies GmbH & Co., KG, Hundsangen, Germany) over a frequency (*f*) range from 1 × 10^–2^ to 1 × 10^6^ Hz. The samples were placed between two stainless steel electrodes (diameter: 15 mm, gap: 0.052 mm) and mounted on a cryostat. During measurement, each sample was maintained under a dry nitrogen gas flow. The temperature was controlled by a Quatro System using a nitrogen gas cryostat, with stability better than 0.1 K. The dielectric measurements of all examined samples were carried out in a wide temperature range (both above and below the *T*_g_) from 173 to 363 K.

### Solubility Studies

Before the dissolution tests, the sample was pulverized using a mortar and pestle and the resulting powder was sifted between 45 and 150 μm screens. The study examined the dissolution rate of itraconazole in a 0.1 M hydrochloric acid solution (pH = 1) at 37 °C using a USP apparatus II PTWS 820D (PharmaTest Apparatebau AG, Hainburg, Germany). Around 300 mg of each sample was placed in 300 mL of medium and stirred at 150 rpm for 120 min. The concentration of the dissolved drug substance was determined by the ultraviolet/visible (UV/vis) method at 255 nm using a T70 UV/vis split-beam spectrophotometer equipped with flow-through quartz cuvettes with a path length of 10 mm (PharmaTest AG, Hainburg, Germany). Measurements were taken every minute until 10 min of the test, then every 5 min, and beyond 60 min every 15 min until the end of the test.

## Results and Discussion

### Innovative Polymeric Materials

First, we briefly outline the motivation and the importance of this study. For many years, PVP has been widely used in the medical, cosmetic, and food industries due to its unique chemical and biological properties: it is nontoxic to humans, biocompatible, and highly water-soluble.^45^ Other properties, such as film-forming ability, chemical and thermal resistance, and its amorphous character, are also desirable and widely utilized in the above-mentioned industrial sectors.^46,47^ Hence, there are numerous works in the literature where the influence of this macromolecule on improving the solubility of APIs by forming ASDs is considered. Unfortunately, despite the great importance of this polymer, scientists still only examine the role of molecular weights (*M_n_*) of commercially available PVP,^48−51^ while the impact of other factors, such as polymer topology or dispersity (*Đ*) on the pharmacokinetic properties of numerous pharmaceuticals, is not described at all. Herein, it should be stressed that this is most likely because the large-scale PVP synthesis is based on an uncontrolled free radical process.^52,53^ As a result, the obtained linear macromolecules consist of both long and short chains, ultimately leading to high *Đ* values (most often above 2, which means poorly defined macrostructural parameters). Moreover, free radical polymerization is mainly employed to produce high-*M_n_* PVP, posing a potential threat of accumulation in the human body or the environment.^54,55^ It should also be kept in mind that for special applications (e.g., in pharmaceutical formulation designing), strict control of polymer parameters is highly required. Therefore, searching for new methods to obtain PVP with precisely desired parameters appears entirely justified.

Herein, it is important to emphasize that although many macromolecules available on the market can improve the pharmacokinetic properties of poorly soluble drugs, PVP appears to be particularly important. The main motivation behind the selection of exactly this polymer for our studies was a unique opportunity to synthesize macromolecules of various topologies and low dispersities, which is far more complicated in the case of other commercially available polymers. Consequently, it was possible to probe the impact of polymer topology on the physicochemical properties, spatial molecular organization, and API solubility in an amorphous solid dispersion.

Recognizing such an extremely intriguing research gap, we have recently developed new polymeric materials with linear (*lin*PVP) and star-shaped (*star*PVP) topologies, employing thermally initiated reversible addition–fragmentation chain-transfer (RAFT) polymerization. Utilizing this method allowed us to gain control over the polymerization process and obtain well-defined polymers characterized by excellent macrostructural parameters in comparison to the commercial sample. Moreover, the chain-end functionalization of polymers synthesized *via* RAFT allows for further modifications of the macromolecules as needed (for example, through copolymerization), contrary to the commercially available PVP (see Figure 1). So far, we have used these PVPs only once to study their influence on the physical stability of amorphous metronidazole and the rate of its recrystallization from ASDs.^56^ Therefore, a highly interesting scientific issue would be to investigate how these innovative self-synthesized materials will affect the properties of a completely different API, namely, ITZ, classified as a liquid crystal, and thereby exhibiting interesting molecular ordering that further influences its solubility. The detailed synthesis procedures, ^1^H and ^13^C NMR spectra confirming the structures of the obtained polymers, and SEC traces allowing the determination of the macrostructural parameters (*M_n_*, *Đ*) can be found in our previous publication.^56^ However, as a reminder, all key information regarding the synthesis of well-defined PVPs with various topologies is also provided in the Supporting Information, SI of this work (see Figures S1–S7 and the description).

### Preparation of ITZ–PVP Binary Formulations and FTIR Studies

First, it is worth noticing that before preparing ASDs with ITZ, we performed cytotoxicity tests on normal human dermal fibroblasts (NHDFs) for both synthesized samples: *lin*PVP and *star*PVP, as well as a reference sample—PVP K30 (see Figure S8 in the SI). The results of these tests, described in detail in the SI, confirmed that the obtained macromolecules exhibit high neutrality and can be successfully utilized as additives in medicinal products. Moreover, terminal groups in the macromolecules obtained via RAFT and star initiator do not affect the biocompatibility of the newly synthesized polymers in any way.

Hence, with the results of cytotoxicity tests, we proceeded to prepare ASDs by the melt-cooling method. Note that a detailed description of this experimental technique can be found in the Materials and Methods Section. The created binary mixtures consisted of ITZ (known for its liquid crystalline character) and three different polymeric materials: (*i*) commercial PVP K30 (*M_n_* = 40,500, *Đ* = 2.05), (*ii*) self-synthesized linear PVP, *lin*PVP (*M_n_* = 37,600, *Đ* = 1.22), and (*iii*) self-synthesized three-armed star-shaped PVP, *star*PVP (*M_n_* = 38,700, *Đ* = 1.48).^56^ While preparing ASDs, we noticed an extremely intriguing relationship. Both linear polymeric matrices (PVP K30, *lin*PVP) are mixed with ITZ in a polymer amount of up to 5 wt %, whereas *star*PVP exhibits a significantly better capability for homogeneous mixing with the API (even up to 15 wt %). At this point, it is essential to emphasize that all used macromolecules have a similar molecular weight (*M_n_* ∼ 40,000). Therefore, the observed effect of better miscibility of *star*PVP with ITZ cannot be attributed to *M_n_* but rather to the topology of the applied macromolecules (see Figure 1). To better understand this phenomenon, infrared measurements were conducted for neat active substance (ITZ) and polymers (PVP K30, *lin*PVP, and *star*PVP), as well as ITZ–PVP 95:5 *w/w* ASDs (see Figure S9 in the SI). Interestingly, no differences in the shape or position of individual bands/peaks throughout the spectral range were observed for neat macromolecules and binary formulations with different PVPs. The obtained results suggest that, in this case, the polymer topology does not affect the type of API–PVP intermolecular interactions. Thus, it can be assumed that the observed differences in miscibility are rather related to how ITZ molecules are localized/distributed along the polymer chains of different matrices, which consequently may lead to differences in improvement in the pharmacokinetic properties of API depending on the type and amount of macromolecule used in binary mixtures.

### Differential Scanning Calorimetry (DSC) Data

**Figure 2:**
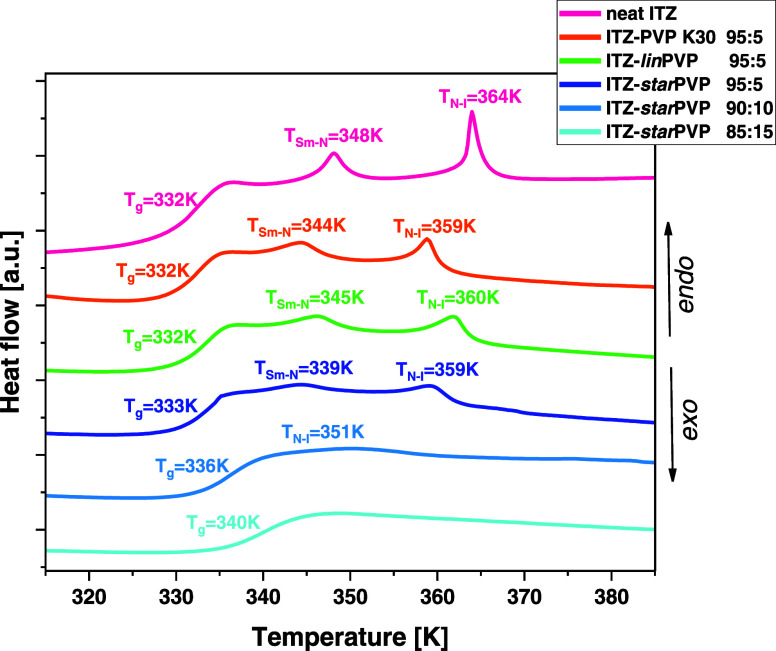
DSC thermograms (ϕ = 10 K/min) collected for neat glassy/amorphous ITZ and ITZ–PVP ASDs with different weight ratios (*w/w*) of both components.

Subsequently, we carried out calorimetric measurements to characterize the thermal properties and phase transitions of both neat substances (ITZ, PVP K30, *lin*PVP, *star*PVP) and API–PVP binary systems. DSC thermograms collected for neat polymers are shown in Figure S10 in the SI, while the data obtained for amorphous ITZ and ITZ–PVP mixtures were compiled in a single graph (see Figure 2). As can be seen, during the heating of neat ITZ (the second scan after initial melting of the crystalline sample and supercooling) at a rate of 10 K/min, three distinct endothermic events are visible. The first phenomenon occurring at the lowest temperature (*T*) is associated with the glass-transition phenomenon (at *T*_g_ = 332 K). Meanwhile, the two remaining endothermic peaks at higher *T* are linked to the LC ordering of ITZ. More precisely, the thermal event at 348 K corresponds to the transition of API from the smectic (Sm) to nematic (N) phase (*T*_Sm-N_), while that at 364 K is related to a transition from the N to isotropic (I) phase (*T*_N-I_). Herein, it is worth noting that the obtained thermogram of neat ITZ and the determined phase transitions are in good agreement with the data reported in previous works.^16,24,26−33,35^

Next, we registered thermograms of ITZ–PVP 95:5 *w/w* ASDs. As can be observed in Figure 2, regardless of the type of polymer applied in the binary system, the *T*_g_ remains unchanged with respect to neat API, while the temperatures of both LC transitions shift toward lower values of *T*: to 339 K (*T*_Sm-N_) and 359 K (*T*_N-I_). It is also worth noting that compared to neat API, the peak intensities representing liquid crystalline transitions have decreased, with the most significant intensity reduction observed for the ITZ–*star*PVP mixture.

As mentioned above, due to the possibility of obtaining homogeneous ITZ–*star*PVP systems with a higher content of the polymer, calorimetric studies were also performed on ASDs, where the weight ratios of API to PVP were 90:10 and 85:15, respectively. The analysis of the obtained thermograms showed that in the case of the ITZ–*star*PVP 90:10 *w/w* mixture, there is a distinct glass transition, but it is slightly shifted toward higher *T* (*T*_g_ = 336 K) due to the higher polymer concentration in the sample. What is particularly interesting, the Sm–N transition is completely suppressed, while the N–I transition has very low intensity and is strongly shifted toward lower *T* (*T*_N–I_ = 351 K). For the ITZ–*star*PVP 85:15 *w/w* system, only one transition corresponding to the glass-transition phenomenon is visible at *T*_g_ = 340 K. In turn, both LC transitions are completely suppressed. It means that the ITZ–*star*PVP 85:15 *w/w* mixture is fully amorphous, which is not possible to achieve in the case of binary systems containing linear polymers (PVP K30 and *lin*PVP); see Figures 2 and S11 in the SI showing DSC curves for ITZ–PVP K30 and ITZ–*lin*PVP, 85:15 *w/w* mixtures. The data presented in the SI clearly demonstrated that the increasing content of *lin*PVP and PVP K30 does not change the *T*_g_ of the whole system, indicating the lack of homogeneity of the binary mixtures for this composition. Hence, the novel star-shaped PVP material appears to be unique compared to both linear polymers. It should be stressed that the obtained results seem to be among the few, where such a small macromolecule content (even 5 wt %) significantly suppresses LC transitions. Moreover, the newly obtained star-shaped macromolecule (unavailable commercially) effectively damps the Sm–N and N–I transitions even at a 15 wt % concentration, imparting a fully amorphous character to ITZ.

It should also be mentioned that we carried out additional DSC measurements with lower and higher ϕ values (i.e., 5 and 20 K/min) on the studied systems. As expected, subtle shifts in the temperatures of all three phase transitions (*T*_g_, *T*_Sm-N_, *T*_N-I_) toward higher values were observed with increasing heating rates (see Figure S12 in the SI).

### X-ray Diffraction (XRD) Data

**Figure 3:**
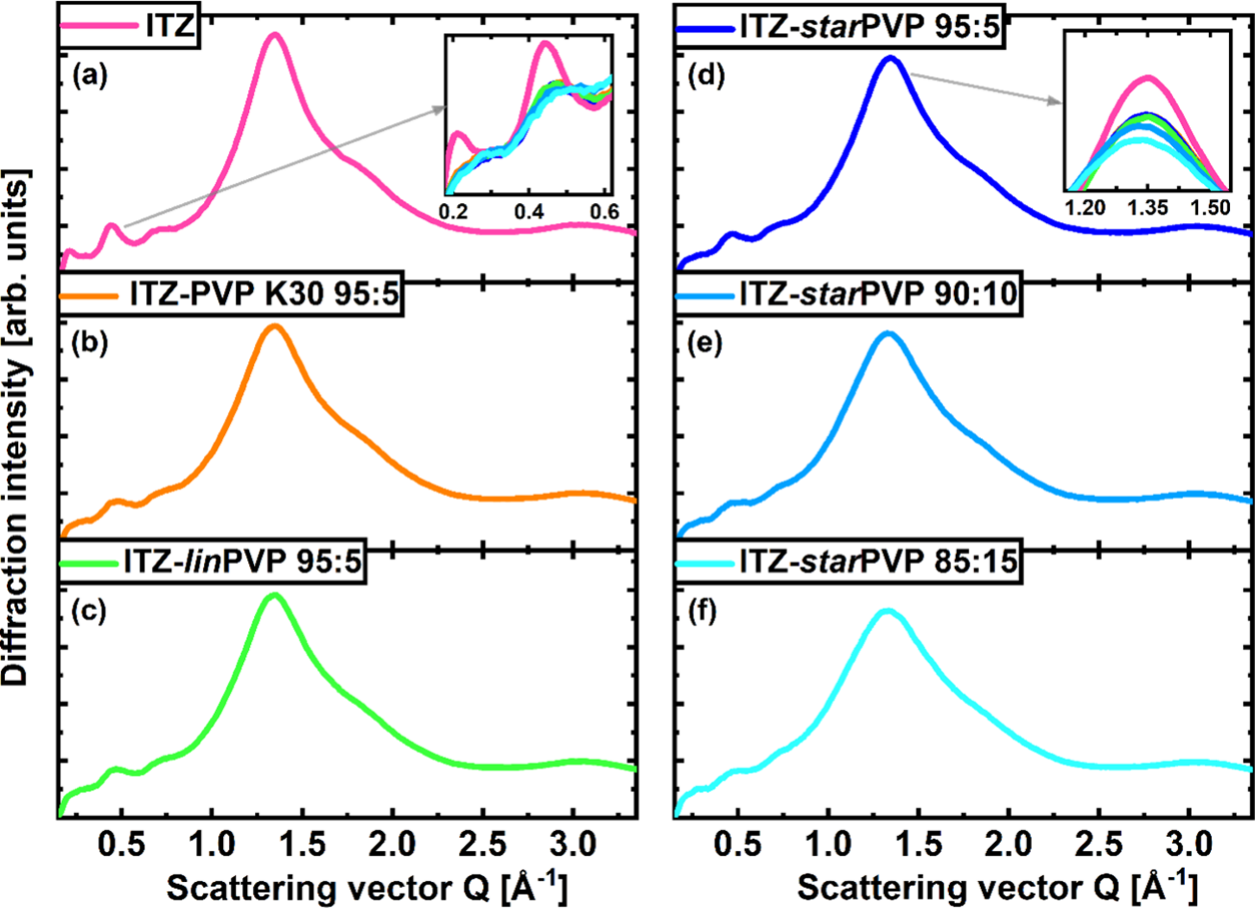
Diffraction patterns of (a) neat ITZ glass and indicated ASDs, (b) ITZ–PVP K30 95:5 *w/w*; (c) ITZ–*lin*PVP 95:5 *w/w*; (d) ITZ–*star*PVP 95:5 *w/w*; (e) ITZ–*star*PVP 90:10 *w/w*; and (f) ITZ–*star*PVP 85:15 *w/w*. The first inset (in panel a) shows the zoomed-in view of the compared diffractograms in the small-*Q* range, while the second inset (in panel d) presents the comparison of the main diffraction peak amplitude.

Noticing exceptionally intriguing results from calorimetric studies, we decided to perform further structural investigations of neat ITZ and analogous ITZ–PVP binary systems (the diffraction patterns of neat PVP polymers and ITZ–PVP K30 and ITZ–*lin*PVP, 85:15 *w/w* ASDs can be found in the SI). As illustrated in Figure 3, XRD patterns of ITZ and its ASDs with various PVP (95:5 *w/w*) samples show characteristic peaks in the small-*Q* range related to the LC order. Generally, in the case of API molecules arranged in Sm layers, a sequence of diffraction peaks at *Q* ∼ 0.2, 0.4, and 0.6 Å^–1^ is usually reported.^30,58^ For the N phase, these peaks are heavily suppressed. Here, significantly higher amplitudes of these small-*Q* peaks are visible for the neat ITZ sample compared to those of ASDs. This can be seen better in the inset in Figure 3a, where all diffractograms were set together. It should be pointed out that the presence of PVP does not practically influence the diffraction data of ASDs in this small-*Q* range. Thus, the suppression of Sm order in these binary systems seems to be due to changes in the ITZ structure caused by the polymer. The inset in Figure 3d shows that also the main diffraction peak of ITZ gets smaller by the presence of PVP, indicating that the nearest-neighbor organization of molecules is also more disordered. However, no big differences in the XRD patterns for various ITZ–PVP 95:5 *w/w* systems were noted. Nevertheless, the data for ASDs of API with varying wt % of *star*PVP demonstrate that with increasing polymer content, there is an evident suppression and a slight shift of the main diffraction peak toward lower *Q*-values (see the inset in Figure 3d), indicating a stronger disordering of the intermolecular structure of ITZ. Moreover, the low-*Q* peaks are smaller in 90:10 *w/w* ASD, suggesting that only the N order is preserved in this system, while in the 85:15 *w/w* mixture, the low-*Q* peaks practically totally disappear, so the structure of ITZ in this system resembles that of isotropic liquid.

Herein, it should be mentioned that additional XRD studies carried out for ITZ–PVP K30 and ITZ–*lin*PVP at 85:15 *w/w* ASDs confirmed the results of DSC measurements, indicating that these binary systems in contrast to PVP–*star*PVP 85:5 *w/w* ASD are nonhomogeneous. For more details, see Figure S13 and the description given in the SI.

**Figure 4:**
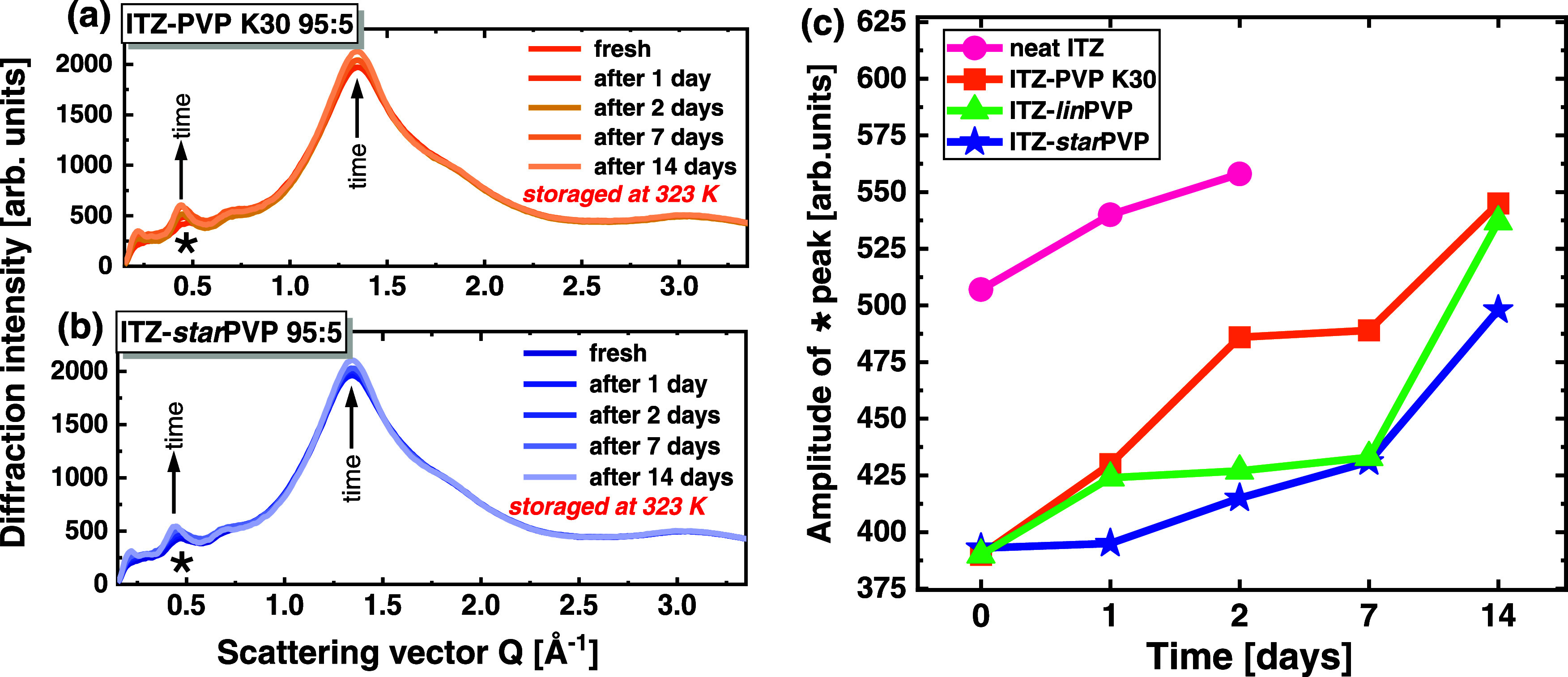
Temporal evolution of the diffraction patterns for (a) ITZ–PVP K30 and (b) ITZ–*star*PVP 95:5 *w/w* systems. The peak at *Q* ∼ 0.47 Å^–1^ marked with an asterisk * was taken for the quantification of the degree of the smectic order. Panel (c) presents the temporal evolution of the amplitude of the * maximum at *Q* ∼ 0.47 Å^–1^ for all studied samples.

Additionally, the differences between ITZ and various PVP 95:5 *w/w* systems were observed for the samples stored at 323 K, which is slightly below the *T*_g_. Panels (a and b) in Figure 4 show the temporal evolution of the XRD patterns for the stored ITZ–PVP K30 and ITZ–*star*PVP 95:5 *w/w* ASDs, respectively. Similar data were collected for neat ITZ and the ITZ–*lin*PVP 95:5 *w/w* system. The results of these long-term structural studies indicated a gradual recovery of the Sm order in time for all samples. The degree of this order was quantified based on the amplitude of the peak maximum located at *Q* ∼ 0.47 Å^–1^ (marked with an asterisk *), and the changes in the amplitude of this peak in time are presented in Figure 4c. The neat ITZ recrystallized after 2 days. Therefore, the data points for the neat API were limited to 2 days. In turn, the ITZ–PVP 95:5 *w/w* ASDs exhibited a gradual recovery of the Sm order with time, manifested by an increase in the amplitude of the * peak and no sign of recrystallization over 14 days. Interestingly, the rate of this structural transformation was different depending on the polymer—the ITZ–PVP K30 system was the least stable, and after 14 days, it recovered almost the same amplitude of the diffraction peak as neat ITZ after 2 days. Meanwhile, the ITZ–*star*PVP ASD was the most stable and had a lower amplitude of this peak after 14 days compared to the ITZ–PVP K30 and ITZ–*lin*PVP systems. The variations in the peak amplitude with time were also accompanied by a shift of the peak position toward *Q* ∼ 0.45 Å^–1^, so toward the position where this peak is located for neat ITZ. This suggests that the addition of PVP polymers may tilt the ITZ molecules by an angle with respect to the layer normal. However, the annealing causes returning of molecules to a more parallel alignment to the layer normal. Nevertheless, it should be noted that these results are related to the annealing of neat ITZ and binary mixtures at 323 K. At room temperature, the rate of restoring Sm phase in API is significantly reduced and it takes months or even years to reach the order characteristic for the vitrified ITZ sample.

### Broad-Band Dielectric Spectroscopy (BDS) Data

**Figure 5:**
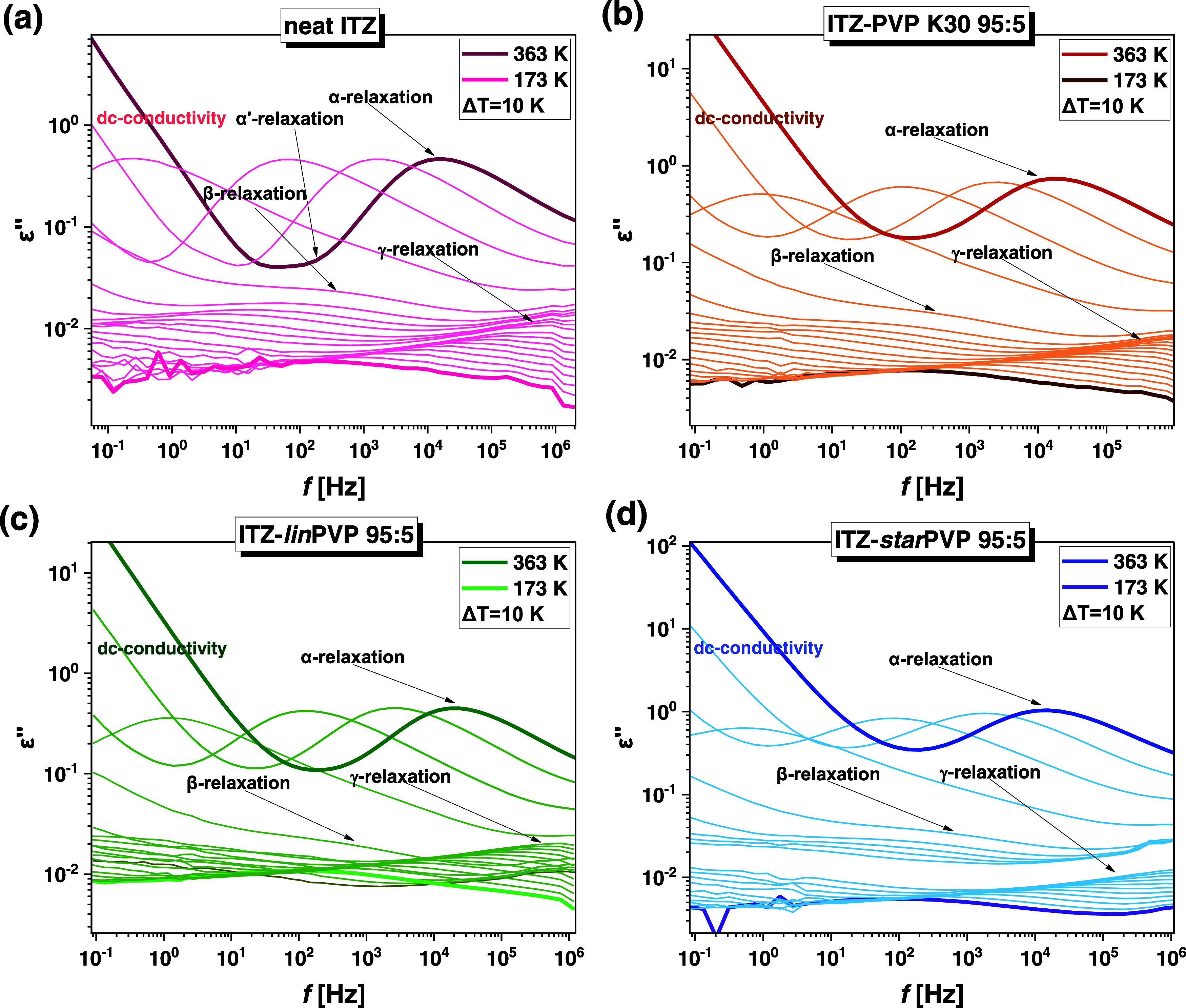
Dielectric loss spectra of (a) neat ITZ^37^ and (b) ITZ–PVP K30, (c) ITZ–*lin*PVP, (d) ITZ–*star*PVP 95:5 *w/w* binary mixtures.

Having the results from DSC and XRD experiments, we carried out molecular dynamics studies of ITZ–PVP ASDs using the BDS method. Dielectric data for neat API were taken from our previous paper.^37^ The measurements were conducted at ambient pressure over a wide range of temperatures, both above and below the glass-transition temperature (*T*_g_). The spectra of dielectric loss obtained for neat API and all API–polymer 95:5 *w/w* ASDs are presented in Figure 5. Note that BDS spectra were also collected for the ITZ–*star*PVP 85:15 *w/w* mixture (see Figure S15 in the SI). As can be observed in Figure 5, at *T* > *T*_g_, two well-visible processes, which shift toward lower *f* with decreasing *T*, can be identified in the spectra of each examined sample. The first one is the direct-current (dc) conductivity associated with the transport of ionic impurities, which are always present in the liquid. Meanwhile, the second process located at higher frequencies (*f*) is the structural relaxation (α) originating from cooperative movements of all molecules in the sample and responsible for the glass transition. Herein, it is worth noting that in the case of ITZ (a compound exhibiting liquid crystalline character), the α-relaxation is associated with complex fluctuations of rotating molecules around its long axis (see Figure 1a, blue arrow). Moreover, in the spectra of neat API (Figure 5a), at *f* lower than those where the α-process occurred, an additional dielectric response, called the α′-process or flip–flop rotation, can be observed. According to the literature data, this relaxation mode is closely related to the rotational motions of the ITZ molecule around its short axis (see Figure 1a, yellow arrow).^27,37,57^ Unfortunately, it is not directly discernible in the spectra of each studied ITZ–PVP 95:5 *w/w* ASDs.

In turn, the dielectric spectra of neat ITZ and ITZ–PVP systems measured in the glassy state (*T* < *T*_g_) revealed the presence of two well-separated secondary relaxations, i.e., the slower β and the faster γ. As demonstrated in our previous papers concerning ITZ and its ASDs with various low-molecular-weight excipients (i.e., acetylated maltose and other APIs from konazole groups), the former one (called β) is a Johari–Goldstein (JG) process having an intermolecular origin, while the latter one (called γ) is a non-JG relaxation of intramolecular character, i.e., a non-JG type.^35,37^

**Figure 6:**
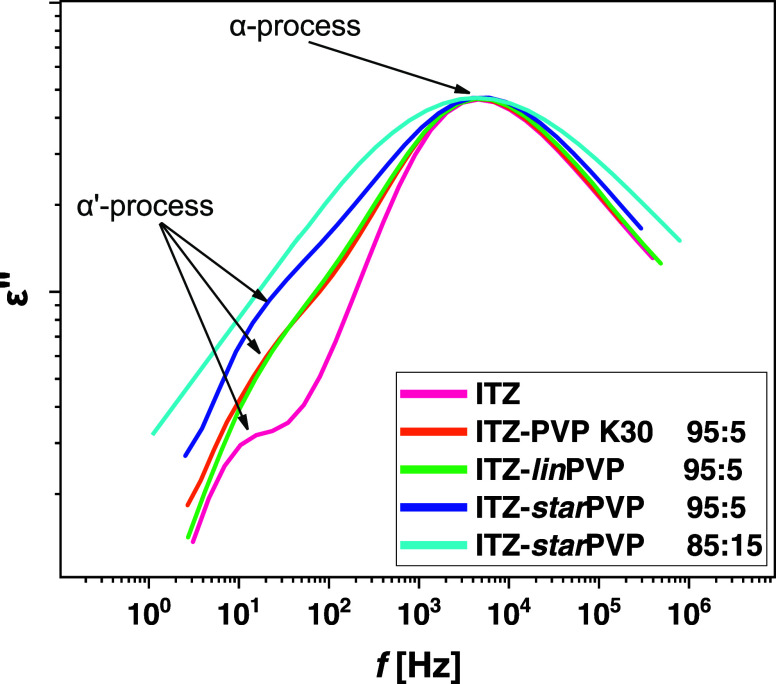
Comparison of dielectric loss spectra measured for neat ITZ and ITZ–PVP binary mixtures (at indicated weight ratios) at *T* = 347 K.

As mentioned above, the α′-process is visible in dielectric spectra of neat ITZ, while for ITZ–PVP 95:5 *w/w* mixtures, this relaxation is not directly observed. To confirm whether the flip–flop rotation (connected directly with the LC ordering in ITZ) indeed occurs in the case of investigated ASDs, we performed the dc*-*conductivity cutting procedure for each sample (also for ITZ–*star*PVP 85:15 *w/w* system) and then compared the shapes of the peaks. The obtained results are presented in Figure 6. As can be seen, for neat API, the α-dispersion is the narrowest and the α′-mode is separated. In turn, for each ITZ–PVP 95:5 *w/w* ASD, the α-peak is significantly broadened and the α′-process is less resolved compared to that of neat API. Importantly, for the ITZ–*star*PVP 85:15 *w/w* sample, the α-dispersion is the widest, and the α′-mode is completely undetectable (see Figure 6, light blue line). This indicates that in all binary formulations, despite the low polymer content (only 5%), there is a strong damping of the liquid crystalline order of ITZ. Furthermore, a complete suppression of both LC phase transitions occurs in the case of the ITZ–*star*PVP 85:15 *w/w* system. Thus, the obtained outcomes of dielectric measurements perfectly correspond to those determined from calorimetric and structural experiments. Herein, it should be noted that a similar scenario, i.e., suppression of LC order by other excipients (acetylated maltose and several APIs: ketoconazole, fluconazole, voriconazole), confirmed also by the decreased amplitude/or even the absence of α′-mode in dielectric spectra, has been reported in our previous works.^35,37^

To get a complete overview of the relaxation processes occurring in all investigated samples (neat API and API–PVP ASDs) and characterize their molecular dynamics, the dielectric loss spectra were analyzed using the Havriliak–Negami (HN) function with an additional term describing the dc-conductivity^59^
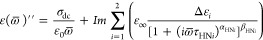
1where σ_dc_ is the dc-conductivity, ε_0_ is the vacuum permittivity, ω̅ is an angular frequency (ω̅ = 2π*f*), ε_∞_ is the high-frequency limit permittivity, Δε is the dielectric relaxation strength, τ_HN_ is the HN relaxation time, and α_HN_ and β_HN_ are the shape parameters representing the breadth and asymmetry of the given relaxation peaks, respectively.

Next, the relaxation times of α- and α′- (*T* > *T*_g_), as well as the β- and γ- (*T* < *T)* processes were calculated from τ_HN_ using the following formula^60^
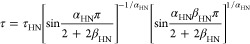
2

**Figure 7:**
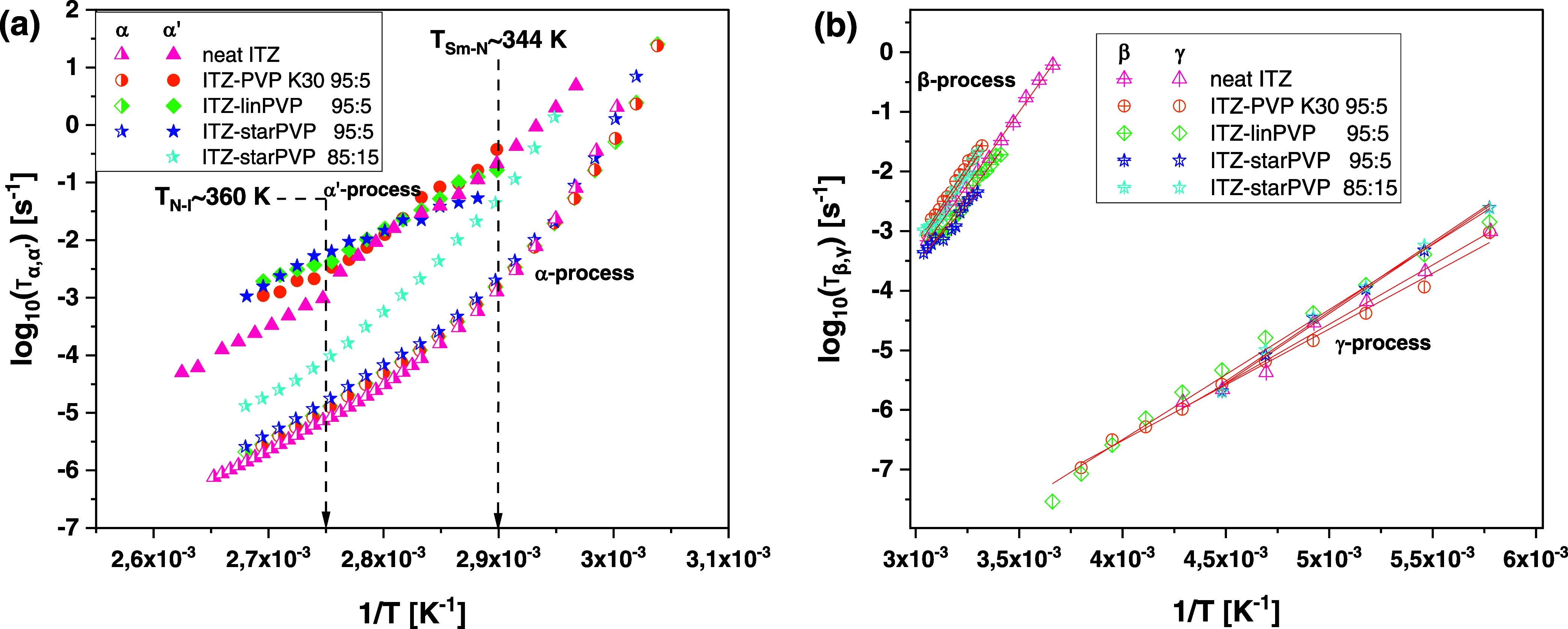
Dependencies of (a) log_10_ τ_α_ (half-filled symbols) and log_10_ τ_α′_ (filled symbols) as well as (b) log_10_ τ_β_ and log_10_ τ_γ_ versus 1/*T* for neat ITZ and ITZ–PVP ASDs with different weight ratios. Solid red lines represent Arrhenius fits. The data for neat ITZ, shown in both panels, were taken from ref. (37)

Figure 7a presents τ_α_ and τ_α′_ plotted as functions of the inverse temperature for neat API and investigated ITZ–PVP ASDs. Interestingly, one can notice clear changes in the dependence of τ_α′_ versus 1/*T*, which are evident at temperatures close to the two LC phase transitions (Sm–N and N–I; see dashed black lines in Figure 7a). It indicates a strong disruption of the molecular order of ITZ, to which the primary α′-process is exceptionally sensitive. However, regardless of the type of polymer used, α-relaxation times for each ITZ–PVP 95:5 *w/w* system and their temperature dependences are very similar and differ only slightly from those determined for neat API. This is in accordance with calorimetric data showing the same values of *T*_g_ (= 332 K) for neat ITZ and 95:5 *w/w* ASDs. It should also be observed that there is a divergence between τ_α_ vs 1/*T* plots for the ITZ–*star*PVP 85:15 *w/w* formulation compared to other, i.e., 95:5 *w/w* mixtures. The reason for that is a difference in *T*_g_ (see Figure 2, Δ*T*_g_ = 8 K) for these samples.

We also characterized the molecular dynamics of neat ITZ and its ASDs with various PVPs at *T* below *T*_g_. As mentioned earlier, two secondary relaxations (β and γ) can be detected in the dielectric spectra of each examined system (Figure 5). The relaxation times of both processes obtained from the analysis of the loss spectra using eq 1, plotted vs 1/*T*, are shown in Figure 7b. To calculate the activation energy barrier (*E_x_*) for these relaxations, the presented data were fitted to the Arrhenius equation
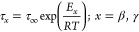
3where *R* is a gas constant. The determined values are listed in Table 1. As can be seen, *E*_β_ for neat API is equal to 93.8 kJ/mol, while for ITZ–PVP systems, it ranges from 76.1 to 103.8 kJ/mol depending on the type of polymer matrix and its content in ASDs. It indicates significant fluctuations in the molecular order of ITZ under the influence of various macromolecules, which consequently affects the dynamics of the slower β-relaxation (a JG type). On the other hand, the activation barrier for the γ-process (a non-JG relaxation) does not show large variations in the studied systems (i.e., *E*_γ_ varies from 35.8 to 44.6 kJ/mol, *E*_γ_ = 38.1 kJ/mol for neat ITZ). It is worth noting that in the case of ITZ–*star*PVP mixtures, the calculated *E*_γ_ was the highest. However, regardless of the weight percentage of the star-shaped polymer in the formulation, it remained unchanged (i.e., *E*_γ_ = 44.6 kJ/mol for ASDs with 5 and 15 wt % of the polymer). It implies that the γ-process is slightly sensitive to the type of macromolecule but not to its content in ASD. In summary, the sensitivity of the β-relaxation to the molecular ordering of ITZ in binary systems is greater compared to that of the γ-relaxation. Herein, it should be recalled that in the case of ASDs of ITZ with other konazoles prepared in various weight ratios, we also observed a similar scenario (i.e., the LC ordering was mainly affected by the dynamics of the β-process, not the γ-mode).^37^

**Table 1:** Values of Activation Energies of β- and γ-Secondary Relaxations Obtained from Dielectric Measurements for Neat API and ITZ–PVP Binary Mixtures

sample	*E*_β_ [kJ/mol]	*E*_γ_ [kJ/mol]
neat ITZ	93.8 ± 1.5^a^	38.1 ± 1.9^a^
ITZ–PVP K30 95:5 *w/w*	103.8 ± 3.1	35.8 ± 1.0
ITZ–*lin*PVP 95:5 *w/w*	90.6 ± 3.0	41.8 ± 1.5
ITZ–starPVP 95:5 *w/w*	76.1 ± 5.2	44.6 ± 1.3
ITZ–*star*PVP 85:15 *w/w*	99.7 ± 4.6	44.6 ± 1.8

a The data were taken from ref. (37)

### Solubility Studies

In the final part of our work, we have conducted solubility studies on neat ITZ, as well as API dispersed in three different polymeric matrices (PVP K30, *lin*PVP, and *star*PVP). The dissolution tests of itraconazole were performed using a hydrochloric acid solution with a pH of 1 as a medium representing the fasted human stomach. It is generally assumed that the gastric emptying time on an empty stomach is usually around 0.5–2 h.^61,62^ Therefore, in our study, we have assumed a measurement time of approximately 120 min.

**Figure 8:**
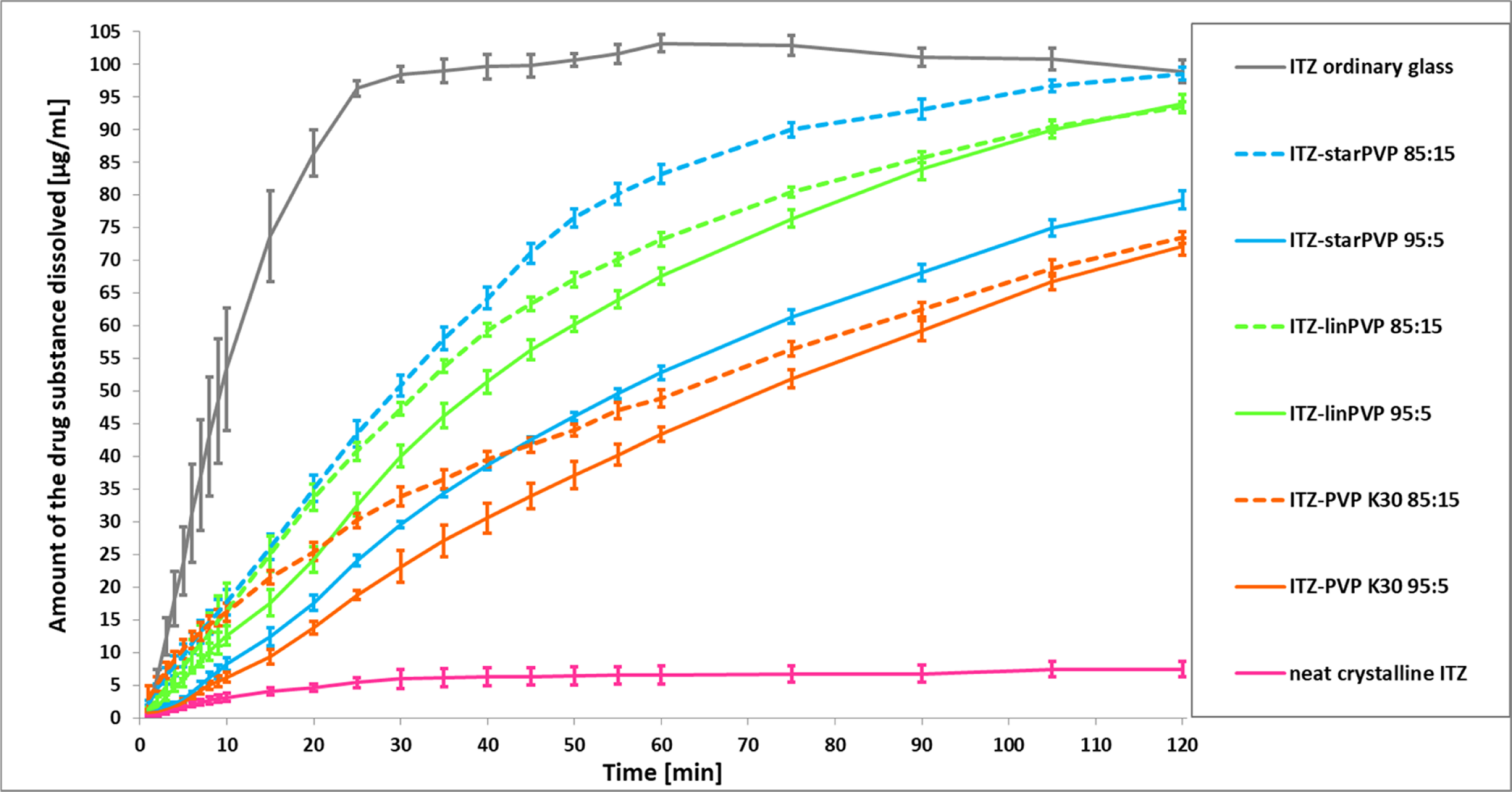
Dissolution kinetics of ITZ and ASDs of ITZ with various PVPs (95:5 and 85:15 *w/w*) in 0.1 M HCl.

The outcomes of the experiments are listed in Figure 8. As can be seen, neat crystalline ITZ exhibits very poor solubility in an acidic medium, at a level of around 5 μg/mL, which is fully consistent with the data reported in the literature.^22,23^ The amorphization, as also indicated in our previous paper,^35^ results in a clear improvement of API solubility (∼100 μg/mL after around 30 min). At this time, the drug substance is rapidly dissolved until a supersaturated state is achieved. However, after about 60 min of the test, the amount of dissolved amorphous ITZ begins to decrease slightly, suggesting the beginning of precipitation (the so-called spring and parachute effect).

For each of the ITZ–PVP systems tested, a significant increase in the solubility of the drug substance compared to that of the neat crystalline form was observed. Moreover, in contrast to amorphous ITZ, the dissolution rate is slower in all binary systems. However, a steady increase in the concentration of the dissolved API is visible over a 120 min period with no signs of precipitation. This supposition finds confirmation in the XRD pattern obtained for the representative 95:5 *w/w* binary system recovered from dissolution measurements. As shown in Figure S14 in the SI, in the tested ASD, ITZ did not recrystallize and, during the dissolution process, the smectic order was restored in the sample.

Interestingly, for ITZ–PVP 95:5 *w/w* ASDs, the greatest improvement in solubility was noted for the API dispersed in *lin*PVP. After approximately 120 min of the experiment, the released API from PVP K30, *lin*PVP, and *star*PVP matrices reached the following concentrations: 70, 90, and 75 μg/mL, respectively. Moreover, even though only *star*PVP exhibits the ability to create a homogeneous mixture with a polymer content of 15 wt %, we decided to prepare 85:15 *w/w* binary formulations containing each examined polymer for solubility measurements. Interestingly, as shown in Figure 8, for ITZ–PVP K30 and PVP–*lin*PVP systems, regardless of the API to polymer ratio, the solubility curves have a very similar course. However, the situation is entirely different for the ITZ–*star*PVP system. Namely, the solubility of ITZ–*star*PVP 85:5 *w/w* (100 μg/mL) is significantly improved in comparison to the same binary systems with 5 wt % of the polymer (75 μg/mL). These observations are related to the fact that the API does not form any LC phases in the ASD with 15 wt % of *star*PVP, which was confirmed before based on outcomes of DSC, XRD, and BDS studies.

The study clearly showed that the choice of polymer type is crucial for improving the solubility of the drug substance. Here, of all of the ASDs tested, the lowest enhancement was determined for the ITZ–PVP K30 systems (95:5 and 85:15 *w/w*), while the best was obtained for the API–*star*PVP 85:15 *w/w* mixture. A probable reason for the highest dissolution rate of API incorporated into a star-shaped PVP compared with linear polymeric matrices is the complete amorphization of the ITZ–*star*PVP 85:15 *w/w* system (the lack of LC ordering). Finally, one should comment on the slowing down of the dissolution rate of ITZ from binary mixtures with respect to neat amorphous API. We suppose that it might be related to the occurrence of some weak intermolecular interactions between both components. Moreover, one can also assume that PVP molecules although well soluble in water may limit access diffusion of solvent to ITZ. Alternatively, the change in the molecular organization of API molecules in the tested systems cannot be ruled out as well. However, these are just hypotheses that should be clarified in the future.

## Conclusions

In this work, calorimetric, diffraction, dielectric, and solubility studies were carried out on ASDs composed of a liquid crystalline API itraconazole and three polymer matrices differing in (macro)structure: commercially available PVP K30, self-synthesized *lin*PVP, and self-synthesized three-armed *star*PVP. Special research attention was paid to examining the impact of polymer topology on changes in the LC order of examined API and the potential improvement of its pharmacokinetic properties. In the initial step of our research, we discovered a highly intriguing relationship. Namely, linear homopolymers (PVP K30, *lin*PVP) exhibited miscibility with the API only at a level of 5 wt %, while the star-shaped PVP mixed with ITZ up to 15 wt %. Calorimetric measurements revealed that each polymer matrix strongly influences both LC (Sm–N and N–I) transition temperatures in ITZ, while the glass-transition temperature remained unchanged compared to *T*_g_ of neat API. Importantly, for each ITZ–PVP 95:5 *w/w* ASD, a significant damping of the mentioned LC transitions was observed. Moreover, ITZ in the ASDs with 10 and 15 wt % of *star*PVP exhibited stronger suppression and destruction of LC order, respectively. Further structural studies confirmed this finding. Moreover, long-term XRD experiments demonstrated the greatest stability of ITZ–*star*PVP systems, indicating the slowest mesophase rebuilding compared with other ASDs. Additionally, dielectric measurements revealed changes in the temperature dependences of τ_α′_ in the vicinity of calorimetric *T*_Sm-N_ and *T*_N-I_, which meant that the α′-process is more sensitive to disruptions in the LC order than the α-one. It was also demonstrated that the fluctuations in molecular ordering affect the dynamics of the slower secondary β-process, while they have practically no impact on the faster γ-mode. Finally, dissolution rate measurements showed that the solubility of all examined ITZ–PVP ASDs in an acidic medium is significantly enhanced compared to neat crystalline API, and, contrary to the amorphous sample, the dissolution curves do not increase as rapidly, preventing saturation and the onset of ITZ precipitation. Importantly, the most significant improvement in solubility was observed for ITZ–*star*PVP 85:15 *w/w*, i.e., approximately 100 μg/mL—representing a 20-fold increase with respect to the neat crystalline API.

It can be assumed that the use of new star-shaped polymeric matrices as novel drug carriers is an interesting perspective. This may contribute to the improvement of ITZ as well as other poorly soluble APIs’ dissolution, ultimately increasing the bioavailability of these drugs. Therefore, from the pharmaceutical sector’s perspective, the results of the performed studies are extremely promising. We believe that they open up a new pathway in scientific discussion regarding the influence of the topology of a relatively well-known polymer, PVP, on the molecular ordering of poorly soluble drugs and the desired improvement in their bioavailability.

## Supporting Information Available

Synthesis of trifunctional CTA, *lin*PVP, *star*PVP, including ^1^H NMR and ^13^C NMR spectra and SEC traces of PVP samples; description of cell culture and cytotoxicity tests; infrared spectra of ITZ–PVP (K30, *lin*PVP and *star*PVP), 95:5 *w/w*, in the amorphous state; DSC data for neat PVP polymers, as well as ITZ–PVP K30 and ITZ–*lin*PVP 95:5 and 85:15 *w/w* systems measured at ϕ = 10 K/min; DSC thermograms collected for amorphous ITZ–PVP 95:5 *w/w* mixtures at ϕ = 5 and 20 K/min; XRD data for neat PVP polymers and nonhomogeneous ITZ–PVP K30 and ITZ–*lin*PVP 85:15 *w/w* mixtures; XRD patterns of ITZ–PVP K30 95:5 *w/w* ASD before and after dissolution studies, and dielectric spectra measured in a wide temperature range for ITZ–*star*PVP 85:15 *w/w* system (PDF)
