# Supplementary material for: The Influence of PVP Polymer Topology on the Liquid Crystalline Order of Itraconazole in Binary Systems
Source: Mol Pharm. 2024 May 17;21(6):3027–39. doi: 10.1021/acs.molpharmaceut.4c00215 (PMC11151211; doi:10.1021/acs.molpharmaceut.4c00215)
Supplement: Supplementary file 1 — mp4c00215_si_001.pdf [file mp4c00215_si_001.pdf]

Supporting Information

The influence of PVP polymer topology on the liquid crystalline order of itraconazole in binary systems

Luiza Orszulak[1]*, Taoufik Lamrani[2], Roksana Bernat[3,4], Magdalena Tarnacka[2], Daniel Żakowiecki[5], Karolina Jurkiewicz[2], Patryk Zioła[2], Anna Mrozek-Wilczkiewicz[2,6], Andrzej Zięba[7], Kamil Kamiński[2], Ewa Kamińska[3]

[1] *Institute of Chemistry, Faculty of Science and Technology, University of Silesia in Katowice, Szkolna 9, 40-007 Katowice, Poland*

[2] *Institute of Physics, Faculty of Science and Technology, University of Silesia in Katowice, 75 Pulku Piechoty 1A, 41-500 Chorzow, Poland*

[3] *Department of Pharmacognosy and Phytochemistry, Faculty of Pharmaceutical Sciences in Sosnowiec, Medical University of Silesia in Katowice, Jagiellonska 4, 41-200 Sosnowiec, Poland*

[4] *Institute of Materials Engineering, Faculty of Science and Technology, University of Silesia in Katowice, 75 Pulku Piechoty 1A, 41-500 Chorzow, Poland*

[5] *Chemische Fabrik Budenheim KG, Rheinstrasse 27, 55257 Budenheim, Germany*

[6] *Biotechnology Centre, Silesian University of Technology, Boleslawa Krzywoustego 8, 44-100 Gliwice, Poland*

[7] *Department of Organic Chemistry, School of Pharmacy with the Division of Laboratory Medicine in Sosnowiec, Medical University of Silesia in Katowice, Jagiellonska 4, 41-200 Sosnowiec, Poland*

**Corresponding author:* luiza.orszulak@us.edu.pl

Experimental

Synthesis of trifunctional chain transfer agent (CTA2)

Trifunctional chain transfer agent (1,3,5-benzyl tri(diethyldithiocarbamate), CTA2) was prepared in a single-step route by the reaction of 1,3,5-tribromomethyl benzene with sodium diethyldithiocarbamate according to the following procedure. In a 100 mL two-neck round-bottom flask equipped with a magnetic stirrer, inlet-outlet nitrogen, and dropping funnel, sodium diethyldithiocarbamate trihydrate (2 g, 8.88 mmol) was dissolved in methanol (40 mL) under a nitrogen atmosphere and cooled to 273 K. A solution of 1,3,5-tribromomethyl benzene (1 g, 2.80 mmol) in methanol (10 mL) was added dropwise over a 30 min period. The reaction was gradually warmed to room temperature and remained under magnetic stirring for a further 72 h. Then, the yellowish precipitate was filtered, washed with cold methanol, and left to dry to constant mass. The structure of the obtained 1,3,5-benzyl tri(diethyldithiocarbamate) was confirmed by ^{1}H NMR spectrum (see **Figure S2**).

Synthesis of PVP with linear topology (linPVP)

Thermally-initiated Reversible Addition Fragmentation Chain Transfer (RAFT) polymerization of N-vinylpyrrolidone (VP) using CTA1 as a chain transfer agent and 2,2'-azobis(2-methylpropionitrile) (AIBN) as an initiator with molar ratios $[VP]_0/[CTA1]_0/[AIBN]_0 = 500/1/0.25$) was carried out as follows. Prior to polymerization, VP was passed through an alumina column to remove the inhibitor. CTA1 (33.5 mg, 0.15 mmol) and VP (8 mL, 74.86 mmol) were placed in a Schlenk flask with a magnetic stirring bar. The solution was purged under nitrogen and purified by three freeze-pump-thaw cycles. Then, the solution of AIBN (187 µL, 0.037 mmol) was added to the reaction mixture and the flask was immersed in an oil bath thermostated at 60 °C to start the reaction. The polymerization was quenched after a predetermined time (t = 3.5 h) by cooling and exposing the reaction mixture to air. The product was precipitated with cold diethyl ether and re-dissolved in chloroform and this cycle was repeated twice. The polymer was isolated, filtered, and then dried under vacuum to a constant mass. The structure of the obtained linear PVP was confirmed by ^{1}H and ^{13}C NMR spectra (see **Figures S3 and S4**). The molecular weight and dispersity of the produced *lin*PVP were determined by size exclusion chromatography (SEC) (see **Figure S7**).

Synthesis of PVP with star topology (starPVP)

Thermally-initiated RAFT polymerization of VP using previously synthesized CTA2 as a chain transfer agent and AIBN as an initiator with molar ratios $[VP]_0/[CTA2]_0/[AIBN]_0 =$

400/5/1) was carried out as follows. Before polymerization, VP was passed through an alumina column to remove the inhibitor. CTA2 (0.13148 g, 0.23 mmol), VP (2 mL, 18.72 mmol) and DCM (4 mL, 200% *v/v* in relation to the monomer) were placed in a Schlenk flask with a magnetic stirring bar. The solution was purged under nitrogen and purified by three freeze-pump-thaw cycles. Then, the solution of AIBN (234 µL, 0.047 mmol) was added to the reaction mixture and the flask was immersed in an oil bath thermostated at 60 °C to start the reaction. The polymerization was quenched after a predetermined time (t = 8 h) by cooling and exposing the reaction mixture to air. The product was precipitated with cold diethyl ether and re-dissolved in chloroform, and this cycle was repeated twice. The polymer was isolated, filtered, and then dried under vacuum to a constant mass. The structure of the obtained three-arm star-shaped PVP was confirmed by ^{1}H and ^{13}C NMR spectra (see **Figures S5 and S6**). The molecular weight and dispersity of the produced *star*PVP were determined by SEC (see **Figure S7**).

Nuclear Magnetic Resonance (NMR)

Nuclear magnetic resonance (^{1}H and ^{13}C NMR) spectra were collected using a Bruker Ascend 500 MHz spectrometer in $CDCl_3$ as a solvent. Standard experimental conditions and the standard Bruker program were used.

Size Exclusion Chromatography (SEC)

Molecular weights (M_n) and dispersity (Đ) of linear and star-shaped PVP homopolymers were determined by size exclusion chromatography (SEC). Viscotek GPC Max VE 2001 and a Viscotek TDA 305 triple detection system (refractometer, viscosimeter and low angle laser light scattering) was used for data collection and OmniSec 5.12 for data processing. Two T6000M general mixed columns were used for separation. The measurements were carried out in DMF/LiBr (0.01M) as an eluent at 303 K with a flow rate of 0.7 mL/min.

Cell culture

Normal human dermal fibroblasts (NHDF) were purchased from PromoCell. The cell line was cultured in Dulbecco's Modified Eagle's Medium (DMEM) supplemented with 15% non-inactivated FBS (*Sigma Aldrich*) and penicillin/streptomycin antibiotics (1% *v/v*; Gibco, Waltham, MA USA). The cells were kept under standard conditions at 37 °C in a humid atmosphere with 5% CO_2 and passed as required by the manufacturer. Additionally, cells were tested against *Mycoplasma* contamination using the PCR technique with specific primers.

Cytotoxicity studies

The cells were seeded in 96-well transparent plates (Nunc, Waltham, MA USA) at a density of 4000 cells per well and incubated for 24 h at 37 °C. Approximately 24 h after seeding, the medium was removed from wells and 200 µL of various concentrations (from 1 to 0.001 mg/mL) of tested compounds (dissolved in DMEM) were added to the plate and incubated for 72 h at 37 °C. Next, compound solutions were exchanged with 100 µL of DMEM without phenol red and 20 µL of CellTiter 96®AQueous One Solution-MTS (Promega, Madison, USA) and incubated for 1 h at 37 °C. After that time, the absorbance of the samples was measured at 490 nm using a multi-plate reader (Varioskan LUX, Thermo Scientific, Waltham, MA USA). The results were normalized to a control consisting of untreated cells. Compounds were tested in triplicate in a single experiment, each repeated three times.

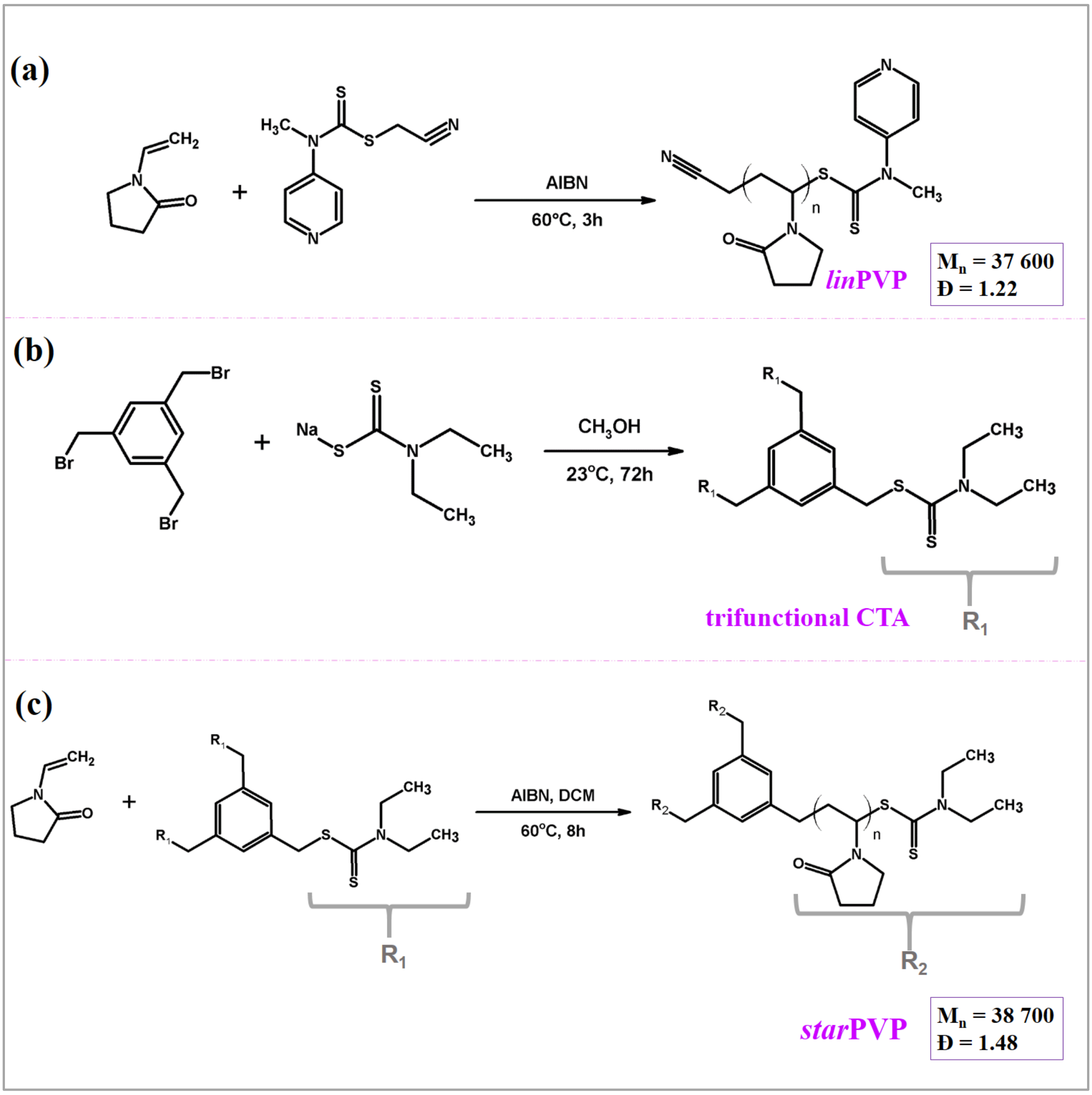

Figure S1. Synthetic pathways for obtaining: (**a**) *lin*PVP, (**b**) trifunctional CTA and (**c**) *star*PVP.

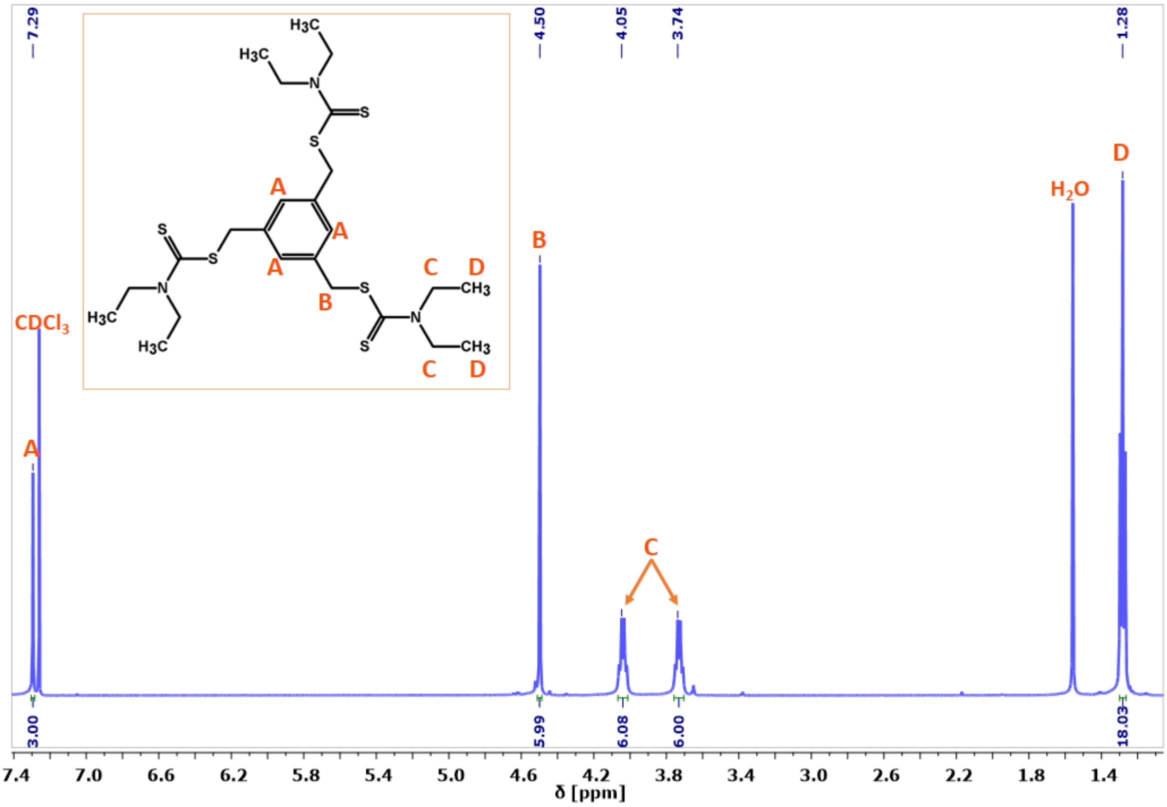

Figure S2. ^{1}H NMR spectrum of trifunctional CTA2 in $CDCl_3$ (500 MHz).

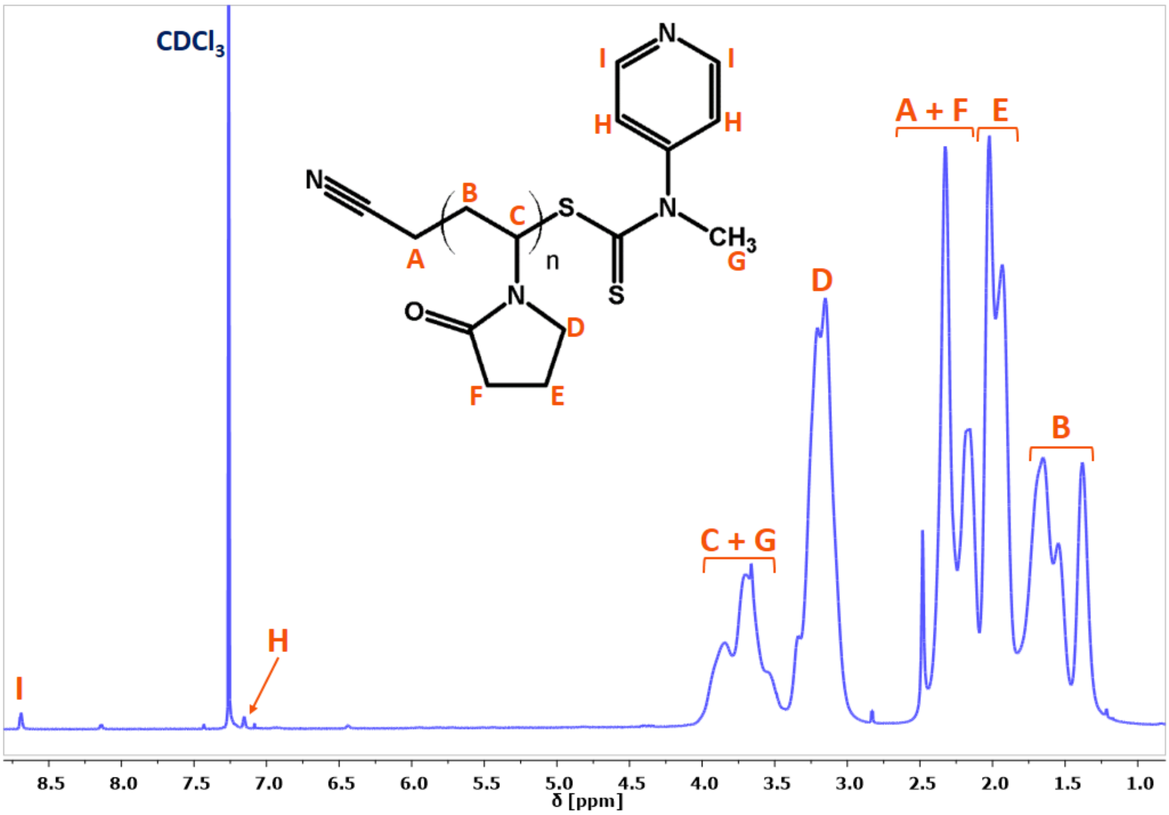

Figure S3. ^{1}H NMR spectrum of *lin*PVP in $CDCl_3$ (600 MHz).

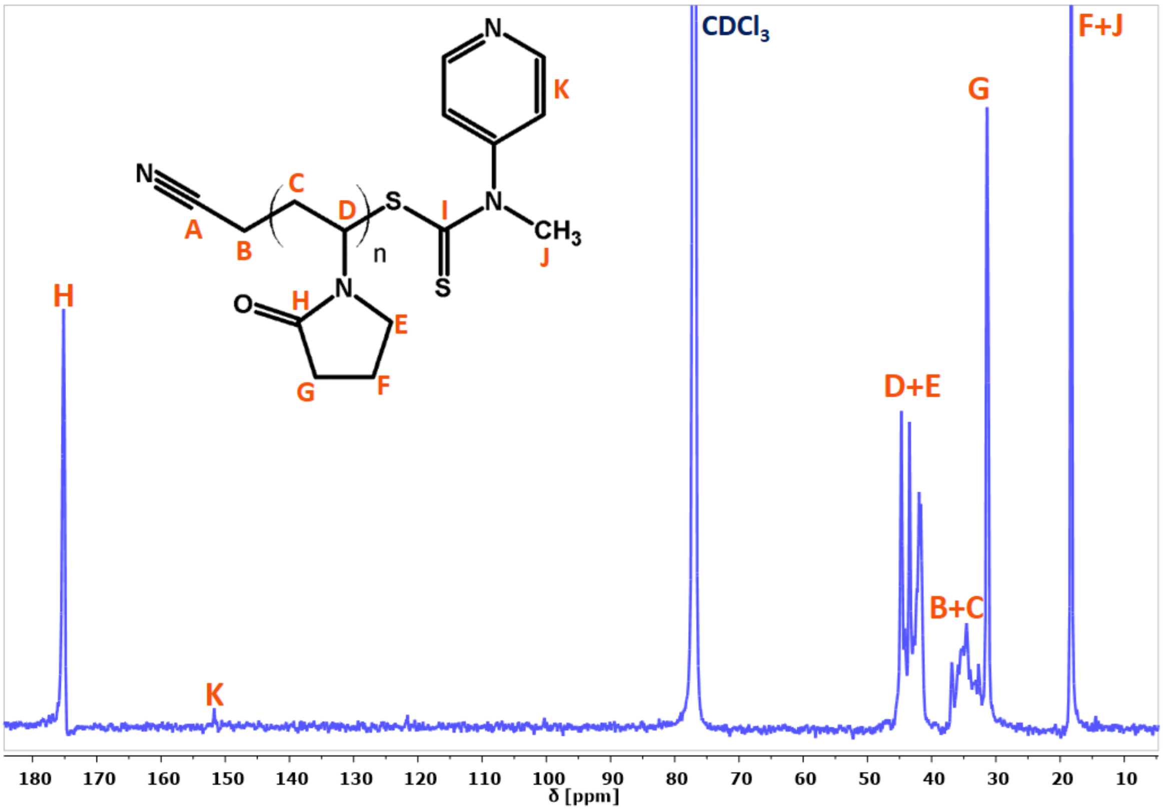

Figure S4. ^{13}C NMR spectrum of *lin*PVP in $CDCl_3$ (600 MHz).

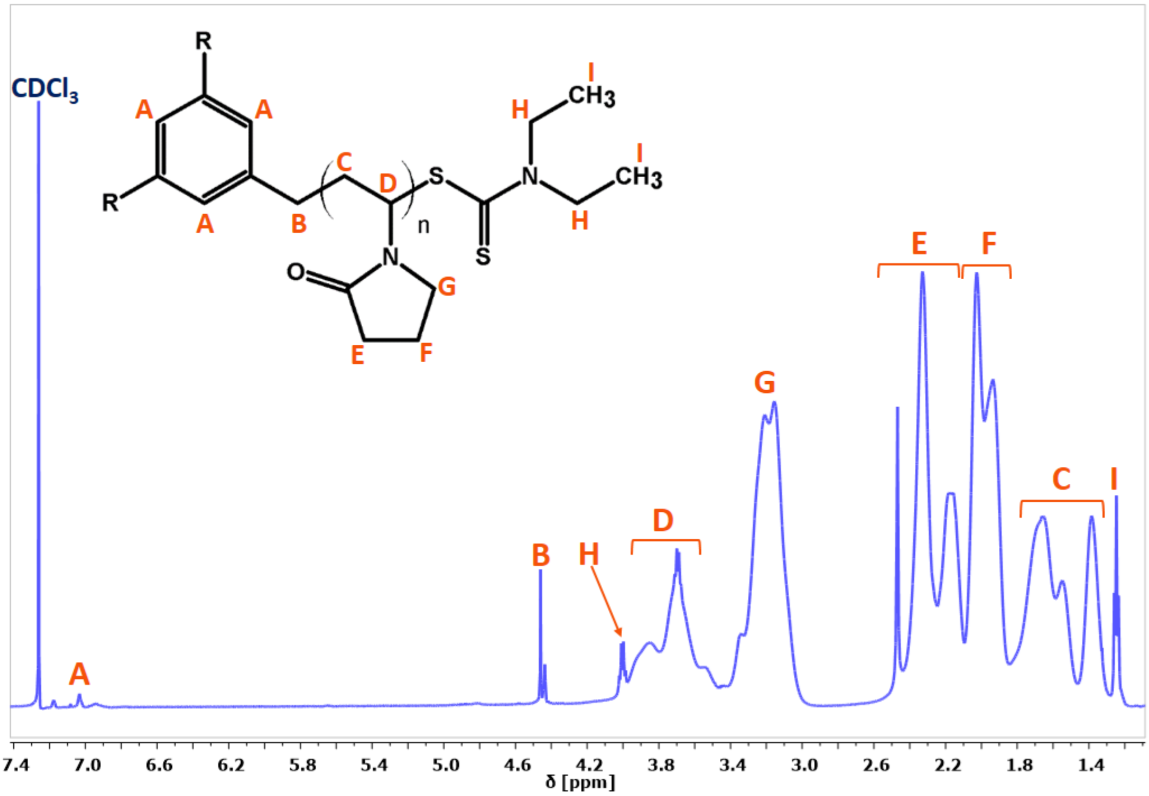

Figure S5. ^{1}H NMR spectrum of *star*PVP in $CDCl_3$ (600 MHz).

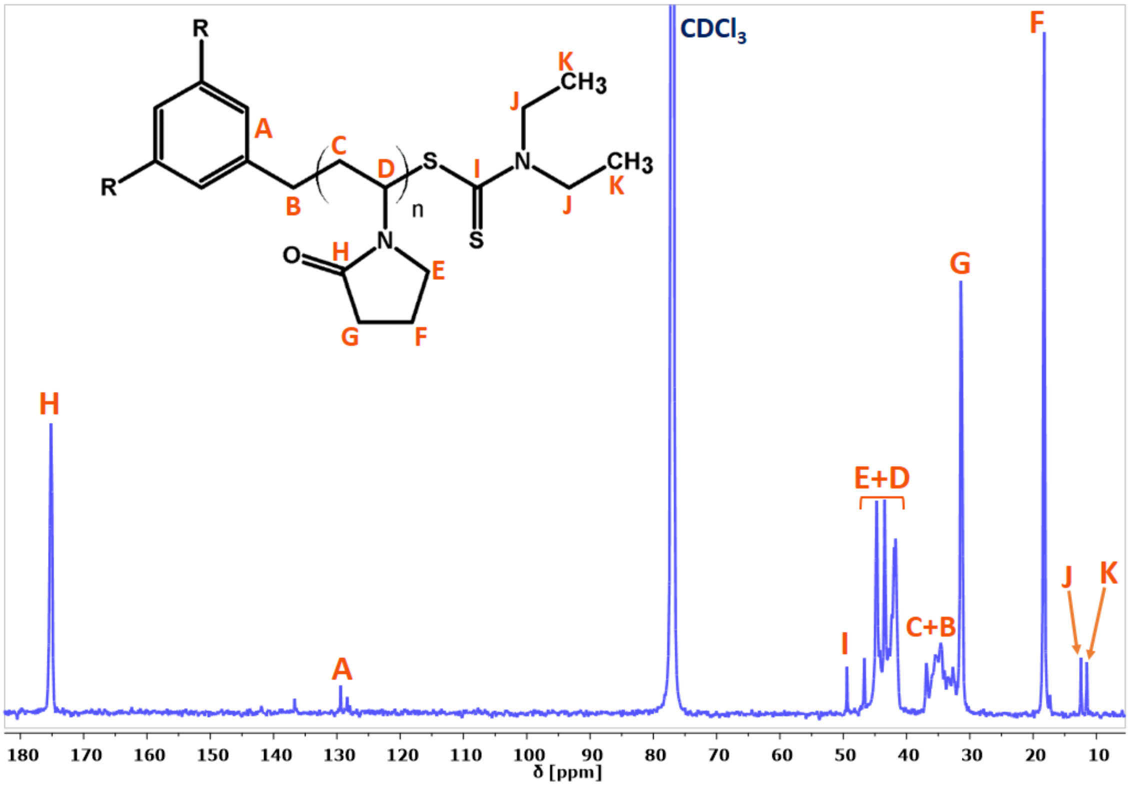

Figure S6. ^{13}C NMR spectrum of *star*PVP in $CDCl_3$ (600 MHz).

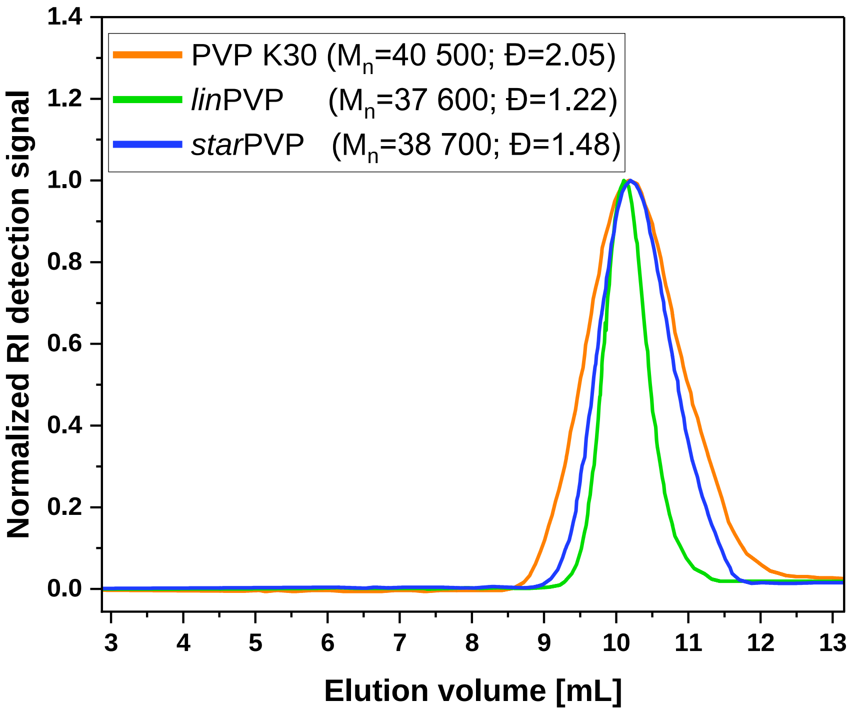

Figure S7. SEC traces of PVP samples: commercial (PVP K30 - orange line) and self-synthesized (*lin*PVP - green line, *star*PVP - blue line).

Results

Cytotoxicity studies

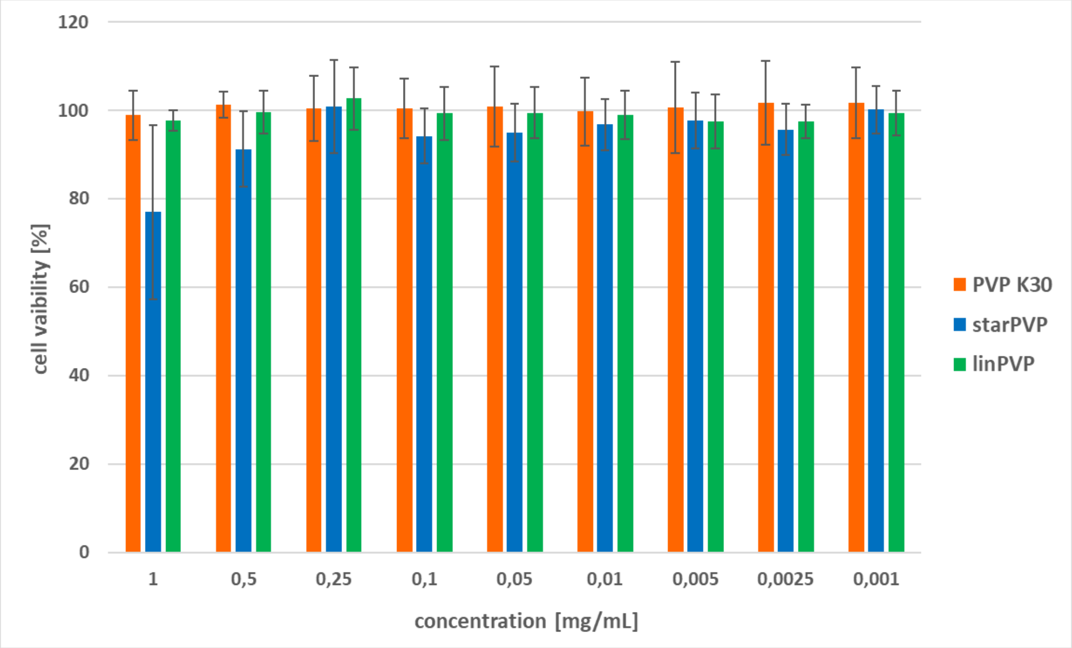

Figure S8. Cell viability seeded onto various PVP samples.

Cytotoxicity tests were conducted for the newly synthesized polymers (*lin*PVP and *star*PVP) prior to the preparation of binary mixtures. The collected data were then compared with the results obtained for PVP K30 (as a reference sample) because it is a fully neutral macromolecule, widely used in the pharmaceutical and cosmetic sectors. As shown in **Figure S8**, the tested compounds exhibit no cytotoxicity towards NHDF cells. The survival fraction in all cases did not drop below 90 %, except *star*PVP, for which a decrease not exceeding 75% was recorded, indicating a neutral impact of the newly synthesized PVP homopolymers on the proliferation of the selected cell line. Herein, it is worth mentioning that we initially selected concentrations for testing that are commonly reported in the literature (i.e., below 0.05 mg/mL). However, upon noticing that the samples showed no cytotoxicity at these concentrations (see our previous paper),[1] we decided to investigate higher concentrations (>0.05 mg/mL). It was revealed that the newly synthesized polymeric materials do not demonstrate harmful effects on the NHDF cell line, even at a concentration of 1 mg/mL.

Infrared data

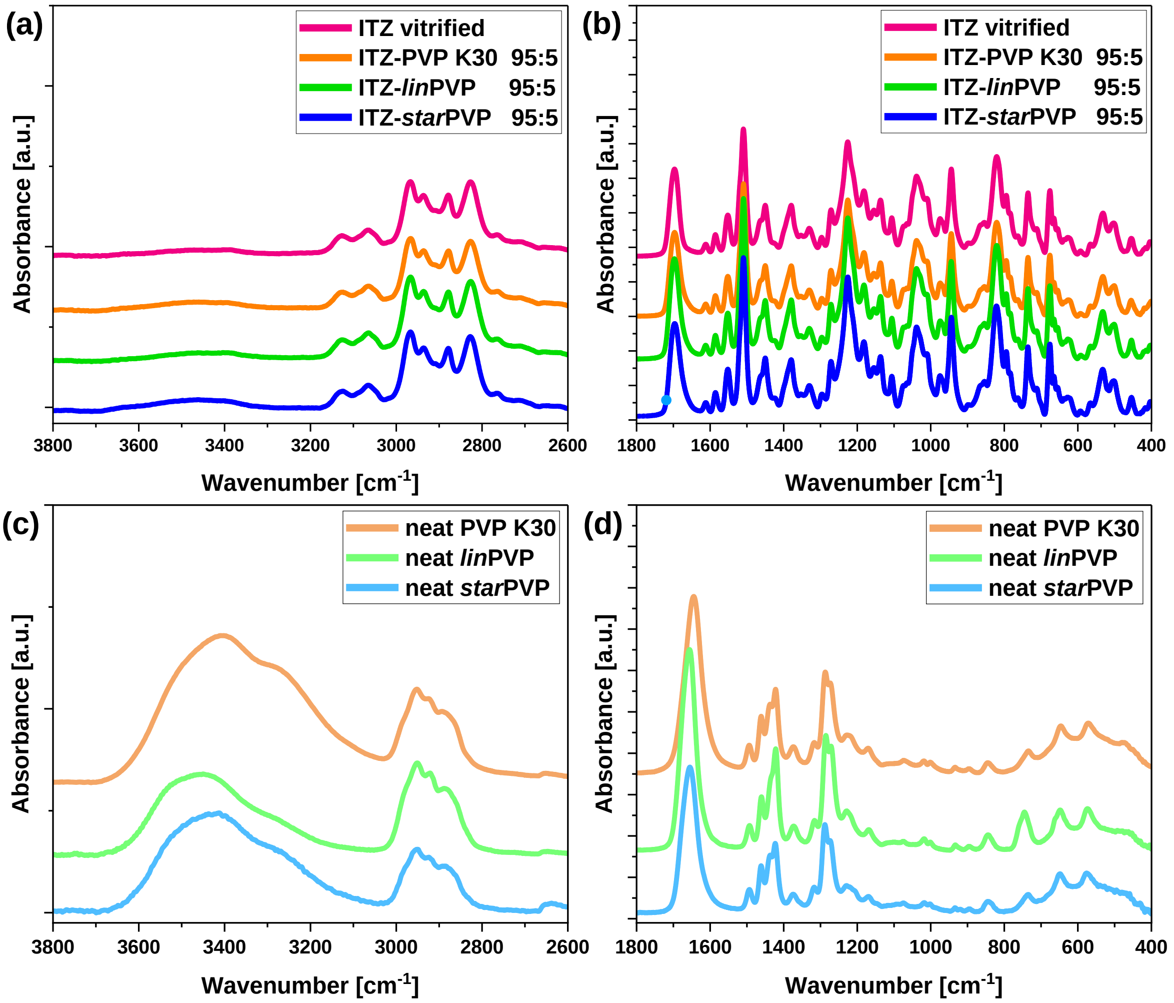

Figure S9. Infrared spectra of binary mixtures of ITZ with three PVP samples (K30, *lin*PVP and *star*PVP) in the weight ratio of 95:5, in the amorphous state, presented in the ranges of **(a)** 3800–2600 cm^{-1} and **(b)** 1800–400 cm^{-1} as well as infrared spectra of neat PVP samples (K30, *lin*PVP and *star*PVP) in the ranges of **(c)** 3800–2600 cm^{-1} and **(d)** 1800–400 cm^{-1}.

DSC data

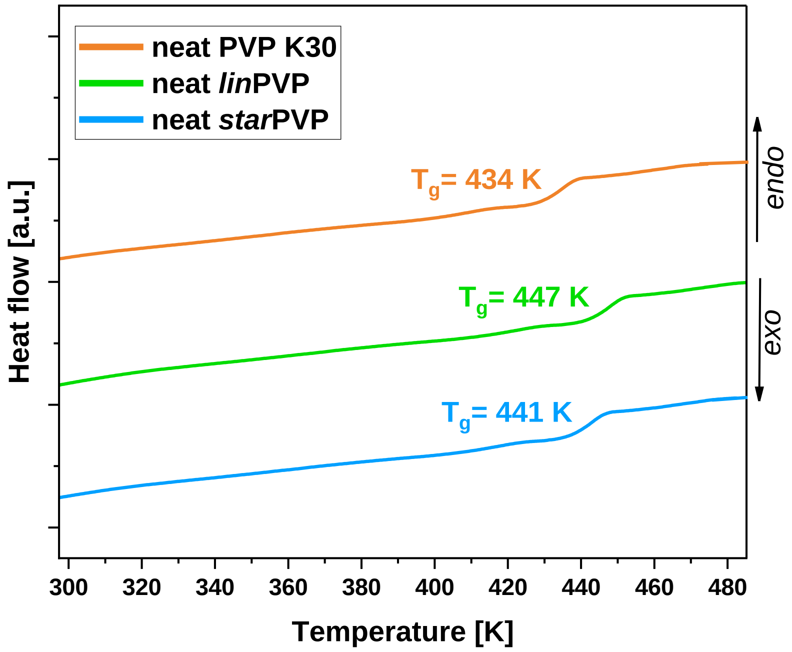

Figure S10. DSC thermograms (ϕ =10 K/min) collected for neat PVPs.

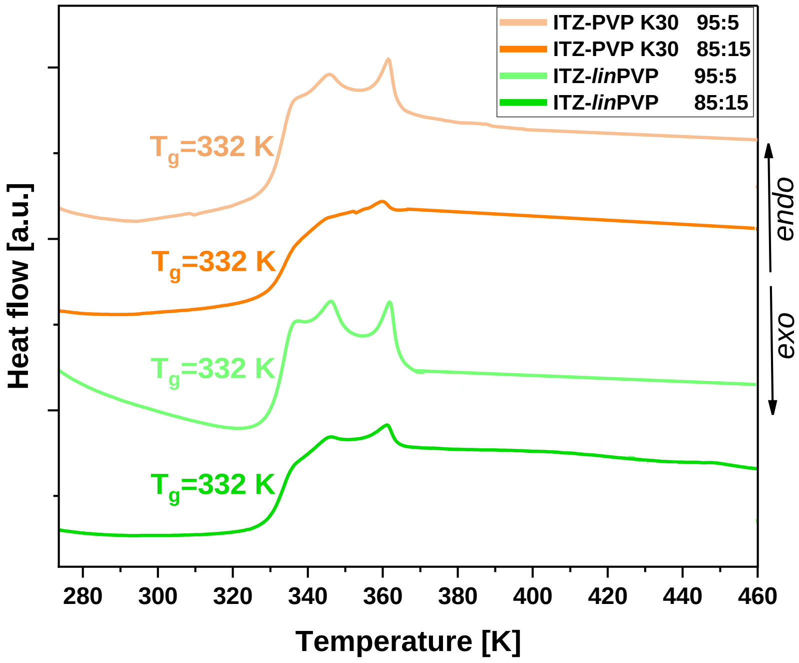

Figure S11. DSC thermograms (ϕ =10 K/min) collected for ITZ-PVP K30 and ITZ-*lin*PVP mixtures prepared at various weight ratios (95:5 and 85:15 *w/w*).

To confirm the limitation in the miscibility of linear polymer matrices with the drug, DSC studies were also conducted for non-homogeneous API formulations with PVP K30 and *lin*PVP (85:15 *w/w*). The collected data were plotted on a single graph alongside their homogeneous counterparts, i.e., 95:5 *w/w* (see **Figure S11**). Analysis of the thermograms obtained for ITZ-PVP K30 and ITZ-*lin*PVP, 95:5 and 85:15 *w/w* systems showed the same glass transition temperatures T_g=332 K regardless of the polymer content in ASDs. It is worth recalling that for ITZ-*star*PVP mixtures, a clear change in phase transition temperatures was detected with increasing polymer matrix content in the binary mixture (the higher the weight fraction of *star*PVP polymer, the higher T_g, **Figure 2** in the main manuscript). Such behavior was not observed in the case of ITZ-PVP K30 and ITZ-*lin*PVP systems, which further reinforces the conviction that there is a limitation in the miscibility of linear polymers with the tested drug at a 5 wt% level. Thus, amongst the tested macromolecules, only *star*PVP exhibits exceptional behavior and miscibility with the API at a 15 wt% level.

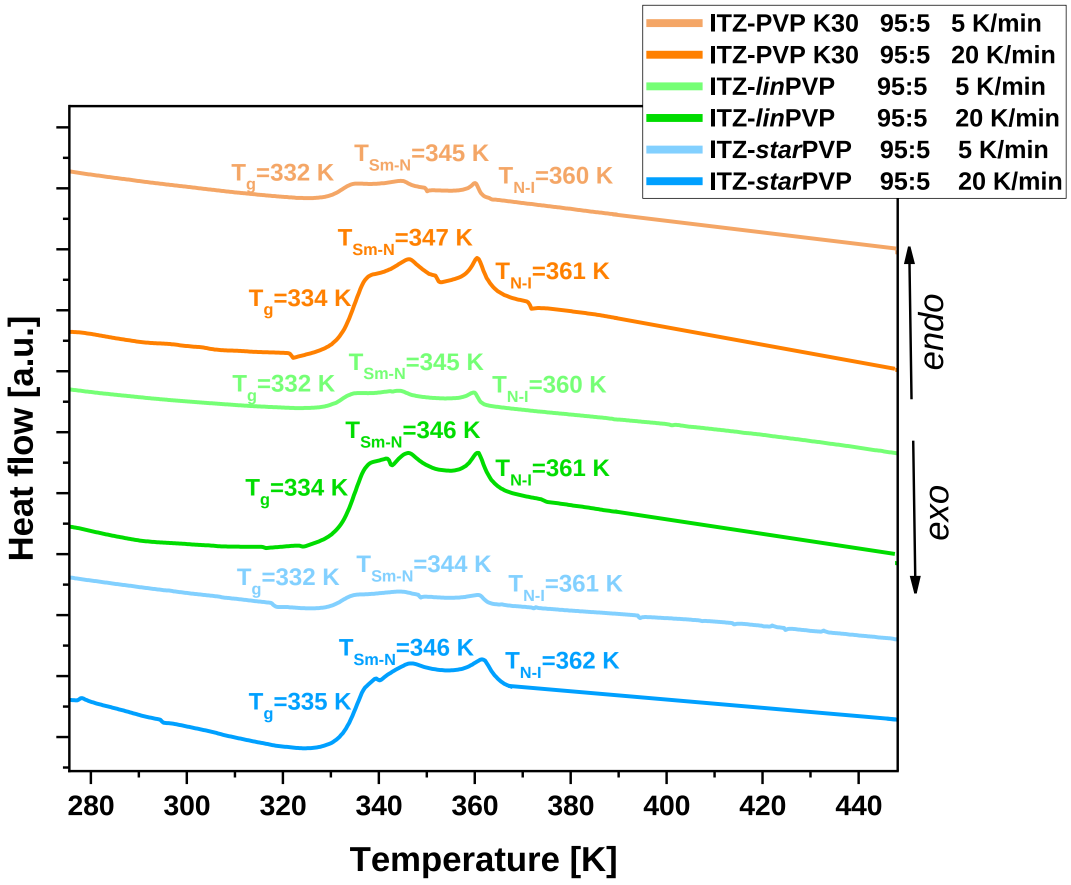

Figure S12. DSC thermograms collected for amorphous ITZ-PVP 95:5 *w/w* mixtures at various heating rates (ϕ =5 and 20 K/min).

X-ray diffraction data

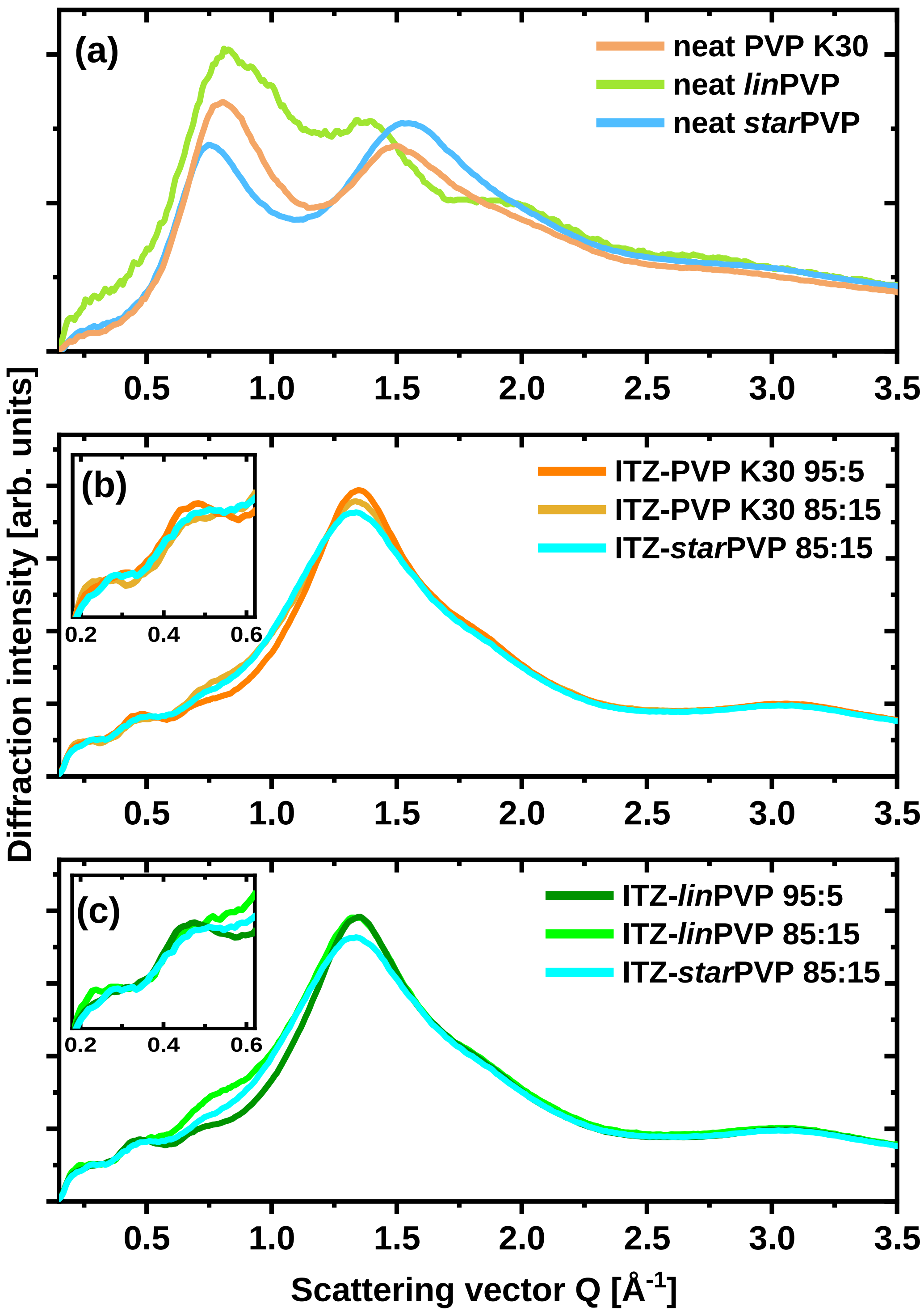

Figure S13. XRD patterns of **(a)** neat PVPs, **(b)** non-homogenous ITZ-PVP K30 85:15 *w/w* compared with its homogeneous counterpart ITZ-PVP K30 95:5 *w/w* as well as ITZ-*star*PVP 85:15 *w/w*, **(c)** non-homogenous ITZ-*lin*PVP 85:15 *w/w* compared with its homogeneous counterpart ITZ-*lin*PVP 95:5 *w/w*, as well as ITZ-*star*PVP 85:15 *w/w*.

In addition to the calorimetric measurements conducted and presented above (see **Figure S11**), we also performed structural investigations for heterogeneous ITZ-PVP K30 and ITZ-*lin*PVP 85:15 *w/w* systems (see **Figure S13**). It turned out that the difference in miscibility could also be successfully observed in the XRD patterns. First, we compared XRD data for neat PVPs. As seen in **Figure S13a**, all utilized polymer matrices distinctly differ in the intensity of the first diffraction peak (around $Q = 0.5 - 1.0$ Å^{-1}). Subsequently, X-ray diffraction measurements were carried out for heterogeneous ITZ-PVP K30 and ITZ-*lin*PVP 85:15 *w/w* systems, and they were compared with their homogeneous counterparts (95:5 *w/w*), as well as with a system showing the highest degree of homogeneous mixing, i.e., ITZ-*star*PVP 85:15 *w/w* (see **Figure S13b**,**c**). As can be observed, for the weight ratio of 85:15, the obtained XRD patterns of the mixtures differ. It is especially noticeable in the range, where PVP exhibits the first diffraction peaks. This peak in the ITZ-*lin*PVP system shows the highest intensity because neat *lin*PVP polymer has the highest peak intensity, indicating that a part of the polymer is not solved in this formulation. On the other hand, the ITZ-PVP K30 85:5 *w/w* mixture exhibits lower intensity in the range of $Q = 0.5 - 1.0$ Å^{-1} compared to the ITZ-*lin*PVP 85:5 *w/w* mixture but slightly higher than the ITZ-*star*PVP mixture because neat commercial PVP K30 has an intensity between linear and star-shaped PVP matrices in this Q range. However, for all investigated mixtures in a ratio of 95:5 (see **Figure 3** in the main manuscript). XRD data show no difference in the analyzed range, confirming the hypothesis that PVP molecules are well miscible with ITZ.

Results of XRD studies obtained for non-homogeneous systems perfectly match the data determined from DSC measurements and additionally confirm the miscibility of ITZ with linear matrices at a weight ratio of 95:5, while ITZ with *star*PVP demonstrates the maximum miscibility at a ratio of 85:15 *w/w*.

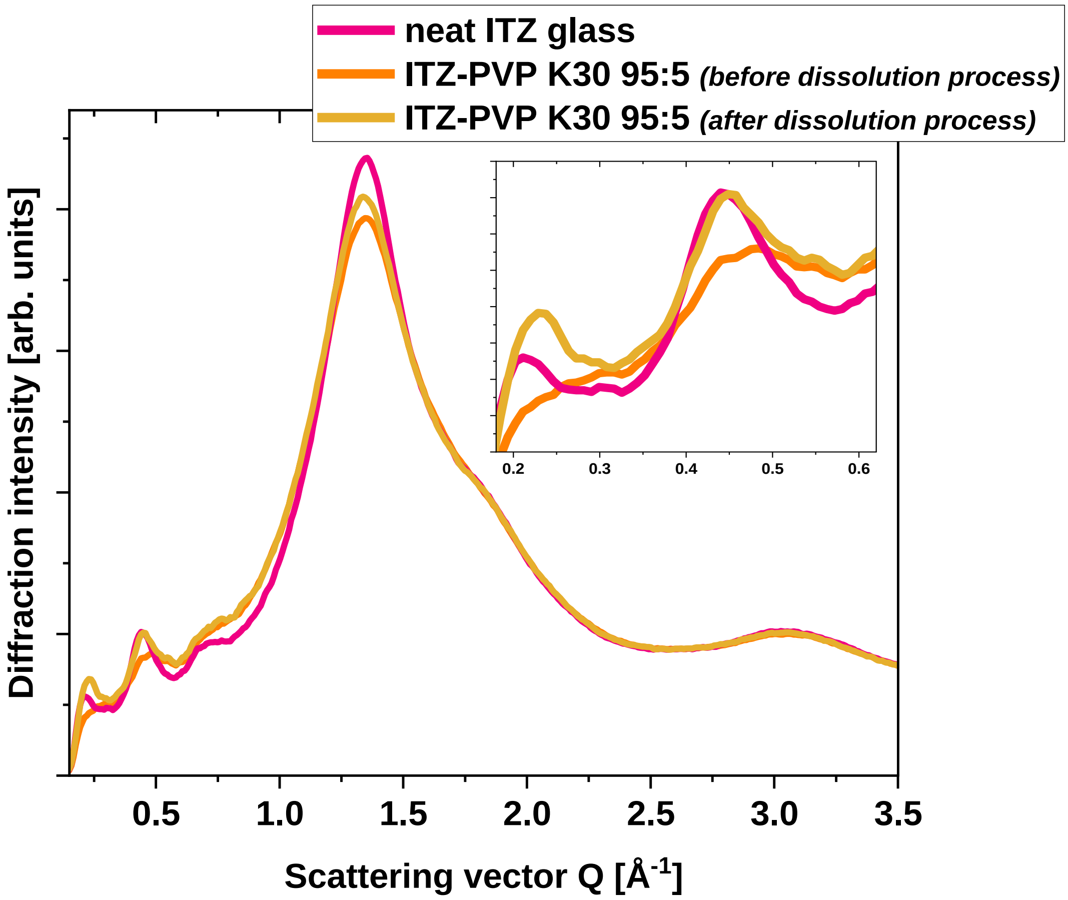

Figure S14. XRD patterns of neat ITZ glass (pink line), as well as ITZ-PVP K30 95:5 *w/w* mixtures before (orange line) and after (yellow line) dissolution process.

Dielectric data

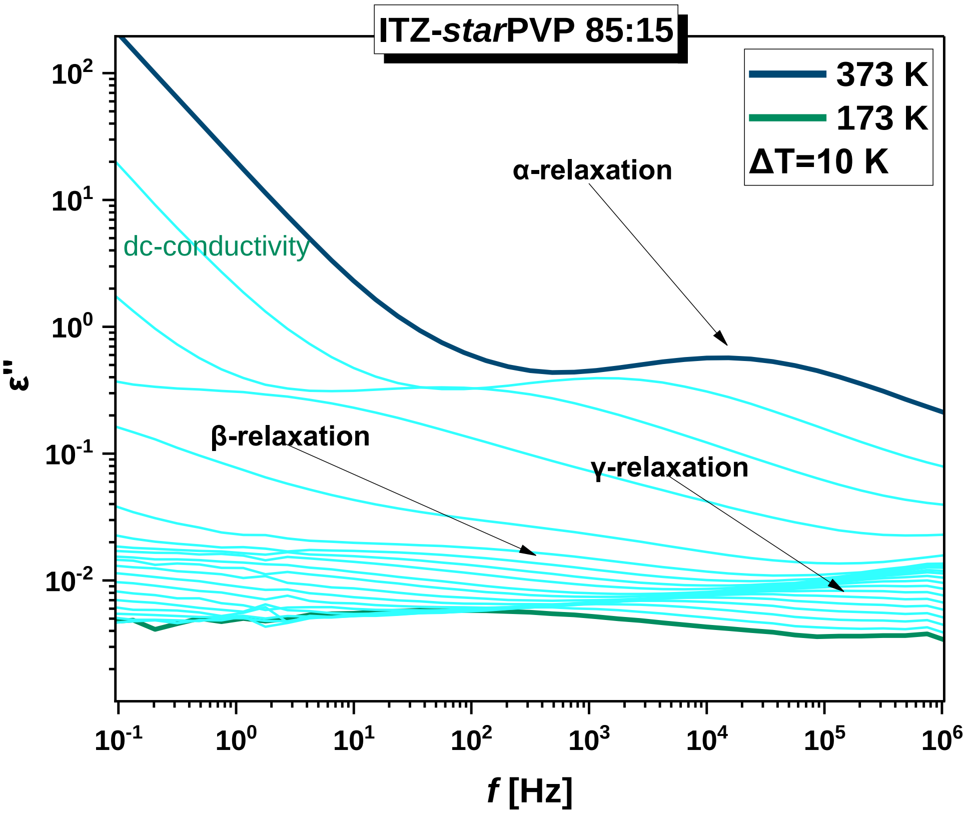

Figure S15. Dielectric loss spectra of ITZ-*star*PVP 85:15 *w/w* mixture.

References:

(1) Orszulak, L.; Lamrani, T.; Tarnacka, M.; Hachuła, B.; Jurkiewicz, K.; Zioła, P.; Mrozek-Wilczkiewicz, A.; Kamińska, E.; Kamiński, K. The Impact of Various Poly(Vinylpyrrolidone) Polymers on the Crystallization Process of Metronidazole. *Pharmaceutics* **2024**, *16* (1), 136. https://doi.org/10.3390/pharmaceutics16010136.
